# Beyond death counts: how COVID-19 affected excess mortality and productive life-years lost across 28 European states, 2020–2023

**DOI:** 10.3389/fpubh.2025.1720864

**Published:** 2026-01-20

**Authors:** Paweł Niewiadomski, Jakub Wojtasik, Błażej Łyszczarz

**Affiliations:** 1 Doctoral School of Medical and Health Sciences, Nicolaus Copernicus University in Toruń, Bydgoszcz, Poland; 2 Statistical Analysis Centre & Doctoral School of Social Sciences, Nicolaus Copernicus University in Toruń, Toruń, Poland; 3 Department of Health Economics, Nicolaus Copernicus University in Toruń, Bydgoszcz, Poland

**Keywords:** COVID-19, Europe, excess mortality, excess years of life lost, excess years of potential productive life lost

## Abstract

**Introduction:**

Excess mortality attributable to COVID-19 has been extensively investigated in the public health literature. This study extends the scope of previous findings by estimating three interrelated measures, excess deaths, excess Years of Life Lost, and excess Years of Potential Productive Life Lost, in 28 European countries across 2020–2023.

**Materials and methods:**

Using annual sex- and 5-year age-specific mortality data for 2002–2019, we applied a multiverse modelling strategy combining all possible baseline windows with four alternative trend specifications. Expected mortality for 2020–2023 was obtained from the best-performing specification, derived using model selection criteria, after trimming outlying forecasts. We define excess mortality burden in terms of three measures: excess deaths, excess Years of Life Lost (eYLL), and excess Years of Potential Productive Life Lost (eYPPLL). Excess mortality was estimated by comparing observed and expected deaths, and was used to obtain eYLL and eYPPLL. Sensitivity analyses included alternative trend assumptions, time-series forecasts, and varying life-expectancy and labour market parameters.

**Results:**

We identified a total of 1,540,034 excess deaths, corresponding to 16,695,365 eYLL and 1,997,095 eYPPLL. Central and Eastern Europe bore the heaviest burden of excess deaths, while the Northern countries were the least affected by COVID-19 mortality. We identified a relatively high health burden in the working population, particularly among men and in Central and Eastern Europe. The male excess in productive life-years lost (eYPPLL) was the strongest among all three measures, further highlighting mortality burden in younger-aged men. These results underscore varying labour market vulnerability to the pandemic across European states.

**Conclusion:**

Our findings underscore marked geographic and sex differences in excess deaths and life-years lost, with particularly high disparities among the working-age population. These patterns emphasise the need for strengthening health systems’ resilience and tailoring post-pandemic recovery efforts to the most affected populations.

## 1 Introduction

Excess mortality attributable to the COVID-19 pandemic is one of the most extensively researched topics in the recent epidemiological literature (see systematic reviews) (1, 2). In general, excess mortality is defined as a residual difference between observed and expected (baseline) mortality, the latter representing a situation had the pandemic not occurred (3–8). The concept attracts interest as COVID-19 mortality statistics have proven to be an unreliable measure of the true pandemic death toll (9, 10). This results from the limited capabilities in identifying deaths that were caused directly by the novel coronavirus, usually leading to an underestimation of COVID-19 mortality (11, 12). Despite its conceptual attractiveness and straightforward interpretation, research in excess mortality estimation encounters challenges. Particularly, methodological choices significantly affect findings, leading to substantially different excess mortality estimates across studies. The choices regarding the mortality measure used, methods and reference periods chosen to determine baseline mortality, and data time units applied lead to meaningfully varying estimates (13, 14).

Yet, despite this abundance of excess mortality estimates, there are still under-researched areas. Particularly, apart from the studies concerned with the pandemic years 2020–21 (11, 14, 15), available findings are usually focused on a single country, and limited cross-country evidence is available to compare the mortality burden across states recently. Also, most previous research reported on mortality in all-age populations or wide age groups (e.g., <65 and 65+) (12). Furthermore, most studies assessed general excess mortality measures of death counts, rates or z-scores (12, 15) and did not contextualise this burden to the wider public health and economic context by providing estimates of years of life or productive life lost.

In this study, we aim to contribute to the literature on excess mortality associated with COVID-19 by focusing on the above under-researched areas. Particularly, we estimate the excess mortality burden in terms of three interrelated measures: excess deaths (eD), excess Years of Life Lost (eYLL), and excess Years of Potential Productive Life Lost (eYPPLL). By estimating eYPPLL, we emphasise how the pandemic eroded the working-age population and labour market potential. This economic view is important because European economies were already strained with labour shortages before COVID-19 (16). Additionally, we provide estimates on 28 European countries [all the European Union (EU) member states and Norway] across the period 2020–2023, allowing for cross-country comparisons both during the pandemic and post-pandemic. Also, in contrast to previous evidence based on provisional mortality statistics, e.g., (17), our analysis relies on the final, complete dataset of death counts by Eurostat. Another value added of this research is relying on disaggregated (5-year) age-group-specific data, allowing for a more nuanced picture of the mortality burden. To reduce arbitrariness in baseline selection, instead of choosing a single reference period, we analysed all possible baseline windows across several trend specifications and applied formal model selection criteria to identify the best-performing forecasting model.

Therefore, this study aimed to assess the excess mortality burden in 28 European countries (EU and Norway) in the period 2020–2023 using sex- and detailed age-specific data from Eurostat. The study used annual death counts for 2002–2019 and implemented a multiverse modelling framework across 18 alternative baseline windows and four alternative trend specifications, trimmed forecasting outliers, and identified the best-performing specification using model selection criteria. This final model was then applied consistently to generate expected mortality for 2020–2023. Excess deaths were then weighted by remaining life expectancy (LE) to compute eYLL and country- and sex-specific work periods to compute eYPPLL. By pairing these two life-year indicators with standard excess death counts, the study reveals not only the scale of premature mortality in Europe but also the magnitude of productive life lost, information essential for understanding post-pandemic labour market pressures and for designing policies that balance health resilience with economic sustainability.

## 2 Materials and methods

### 2.1 General approach

This study used annual all-cause mortality and population data from Eurostat (18–20) on 28 European states (27 EU countries and Norway) to estimate the sex- and 5-year age-group-specific excess mortality burden during the pandemic and post-pandemic years, 2020–2023. We define excess mortality burden in terms of three interrelated measures: eD, eYLL, and eYPPLL. Data from the 2002–2019 period were used to build a set of regression models across multiple baseline windows and trend specifications, from which the best-performing specification was selected and subsequently used to predict expected mortality for 2020–2023. By comparing expected mortality with observed mortality during the period 2020–2023, we identified whether excess health burden was present in particular countries, age and sex groups, and years.

### 2.2 Definition and measures of excess mortality burden

We define excess mortality burden as additional negative mortality effects resulting from the COVID-19 pandemic. The appropriate approach for assessing the excess mortality burden involves accounting for both direct and indirect effects of COVID-19. The former category represents deaths caused by SARS-CoV-2 infection, while the latter, deaths related to delayed access to treatment, e.g., in cancer care (21), emergency or routine care (22), or mortality caused by pandemic-borne increased substance use (23) and suicides (24), among others.

We use three measures of excess mortality burden. The first is the number of excess deaths, defined as the difference between the number of observed deaths and the number of deaths expected (based on historical data) in the absence of the pandemic. This indicator provides a more comprehensive view of the pandemic’s impact beyond reported COVID-19 deaths (25). The second measure of eYLL is based on Years of Life Lost, a public health metric that quantifies the impact of premature mortality by assigning higher weights to deaths at younger ages. It represents the total number of years individuals would have lived if they had not died prematurely (26, 27). Finally, the third measure of eYPPLL is based on Years of Potential Productive Life Lost, an indicator focusing on the economic and societal consequences of premature mortality in the working-age population (28). eYLL and eYPPLL differ from the usual measures of YLL and YPPLL by using input data of excess mortality, instead of regular mortality data.

For the three measures of excess mortality burden, we estimated counts and age-adjusted rates (European standard population) per 100,000 population in each year, country, and sex.

### 2.3 Data characteristics and sources

Annual data on population and all-cause deaths were retrieved from the Eurostat databases (18, 19). COVID-19 deaths data was also retrieved from Eurostat (cause-specific deaths data) (20). Based on the available data, we analysed 28 European countries, including all EU members and Norway. We used country-level data from the 2002–2023 period, broken down by sex, age, and country. The Eurostat mortality database provides death counts for every single age, but due to the small number of cases at young ages in some (low-populated) countries, we aggregated deaths into 18 5-year age groups (0–4, 5–9,…, 80–84, 85+) (15). Mortality data from 2002 to 2019 served as the pre-pandemic baseline for forecasting expected mortality in 2020–2023, using multiple alternative baseline windows.

For some countries, the Eurostat dataset contained missing data points. However, the data completeness was satisfactory, with average population (mortality) data coverage of 99.40% (99.99%), with the lowest value for Hungary (96.89%). In eight out of nine cases, missing entries referred to the population data, mainly in 2002–2010 and age 85+. We imputed missing data using interpolation or applying a trend of the closest age group for which the data was available (for details, see Supplementary file).

### 2.4 Excess mortality estimation

There is no gold methodological standard for mortality predictions, and the choice of method depends on the frequency of observations (from daily to annual), the number of observations used, the length of a reference period, and the specific interest of the research (29). Previous studies proved that estimates can differ depending on the method and reference period (30).

In this study, we aimed to look at excess mortality in more granular age groups than most previous studies, which used either all-age figures combined (31, 32) or wide age intervals, e.g., <65 and 65+ (12) or 0–19, 20–30, 40–64, 65–79, 80+ (33). Our choice was determined by the fact that, apart from estimating excess deaths, we aimed to assess eYLL and eYPPLL, requiring more detailed age-specific data. Unfortunately, this choice comes at the expense of a more aggregated time dimension of data used. Because the weekly death counts for young age groups in low-populated countries are very low and unstable, we used annual data. This approach restricts the selection of estimation methods, as typical seasonal mortality trends are not discernible in annual data. However, previous studies have shown that the excess mortality estimates using weekly and annual data are generally comparable (32, 34).

Among methods applicable to mortality forecasting, we used Ordinary Least Squares (OLS) regression, consistent with previous studies on excess mortality (32, 34–36), with mortality rates (deaths per 100,000 population) regressed on the time variable (year). We did not include age-structure variables because we estimated age-group-specific models. Excess mortality estimates can be sensitive to both the length of the pre-pandemic baseline and the assumed functional form of the time trend. Therefore, to avoid arbitrariness, we analysed all possible consecutive baseline periods ending in the last pre-pandemic year (2019) and estimated a separate model for each. Additionally, for every sex and age group, this multiverse baseline procedure was applied across four alternative model specifications:

- S1—linear trend with full baseline period 2002–2019;
- S2—linear trend with shorter baseline period 2010–2019;
- S3—linear and quadratic trends with full baseline period 2002–2019;
- S4—linear and quadratic trends with shorter baseline period 2010–2019.

For each specification and baseline window, we forecasted sex- and age-specific mortality rates for 2020–2023. Because predictions from very short windows can be unstable, we applied a Tukey 1.5 × IQR (interquartile range) rule to identify outlying forecasts within each specification and year. Outliers were excluded, and the remaining forecasts were averaged within each specification to obtain expected mortality rates for 2020–2023.

To illustrate, under specification S1, for each country, sex and age-group, we estimated all possible OLS models with baseline windows 2002–2019, 2003–2019, …, 2018–2019, and 2019-only, predicted annual values for 2020–2023, and averaged them while excluding outliers (>1.5 IQR from median). The same multiverse procedure was used for S2 to S4 specifications.

Across all countries, sexes and age groups, this procedure yielded 4,032 fitted models (28 countries × 2 sexes × 18 age groups × 4 specifications). To select one forecasting specification to apply uniformly across all strata, we evaluated S1–S4 using pre-pandemic goodness-of-fit criteria: Akaike Information Criterion (AIC), Mean Absolute Error (MAE), and Root Mean Squared Error (RMSE). For each of the 4,032 cases we recorded which specification achieved the best value for each metric, and the specification with the highest overall number of wins across all metrics was selected as the final forecasting model. This approach provides a transparent, data-driven basis for model choice and follows principles of multiverse analysis previously applied in cross-country excess mortality research (30).

Expected deaths for 2020–2023 were derived by multiplying expected mortality rates by observed population counts (25) and excess mortality for 2020–2023 was calculated as the difference between the observed and expected values.

95% confidence intervals for each model were computed using the formula:


$$\begin{array}{l} y_{a,c,s,t}={\hat{y}}_{a,c,s,t}\pm t_{0.975,\;\;n-1}\\ \sqrt{\frac{\underset{i\in \left\{2002\mathrm{,,}..\mathrm{,,}2019\right\}}{\sum }{\left(y_{a,c,i,s}-\hat{y_{a,c,i,s}}\right)}^{2}}{n-1}\left(\frac{1}{n}+\frac{{\left(x_{0}-\bar{x}\right)}^{2}}{\underset{i\in \left\{2002\mathrm{,,}\ldots \mathrm{,,}2019\right\}}{\sum }{\left(x_{i}-\bar{x}\right)}^{2}}\right),} \end{array}$$

where $t_{0.975,\;\;n-1}$ is 97.5% point function of t-Student distribution with n-1 degrees of freedom, ${\hat{y}}_{c,t,a,s}$ is predicted value for country c, year t, age group a and sex s. Aggregated estimates for all ages or both sexes were obtained by summing age-specific predictions. Confidence intervals for these aggregated estimates were computed using standard OLS-based variance formulas under the assumption of independent age-specific errors.

For aggregated estimates (all countries combined, four regions and/or years), we used a confidence interval estimation method based on weighted mean and standard error as widely implemented in meta-analysis techniques. Namely, each of *k* countries contributes an estimate of the effect size $E_{i}$, i∈{1,,…,,k} accompanied by its variance $\mathrm{Var}\left(E_{i}\right)$. The weight assigned to each country is defined as the inverse of this variance: $w_{i}=\frac{1}{\mathrm{Var}\left(E_{i}\right)}$. The pooled effect size $E_{m}$ is then calculated as a weighted average of countries included in the study $E_{m}=\frac{\sum_{i=1}^{k}w_{i}E_{i}}{\sum_{i=1}^{k}w_{i}}$. The variance of the pooled effect is given by $\mathrm{Var}\left(E_{m}\right)=\frac{1}{\sum_{i=1}^{k}w_{i}}$, and the corresponding standard error is $\mathrm{SE}\left(E_{m}\right)=\sqrt{\frac{1}{\sum_{i=1}^{k}w_{i}}}$. To construct a confidence interval around the pooled estimate, one typically uses the normal approximation:


$$\mathrm{CI}=E_{m}\pm Z_{\alpha /2}\cdot \mathrm{SE}\left(E_{m}\right),$$

where $Z_{\alpha /2}$ is the critical value from the standard normal.

### 2.5 Excess life years lost estimation

As for the eYLL estimation, we relied on our excess deaths estimates and used the pre-pandemic 2019 life expectancy (37). Similarly to the population and mortality data used, LE data were divided into 18 age groups. LE for particular age groups was computed using the arithmetic mean, e.g., for the group of females aged 20–24, the average LE was the mean value of LE of women aged from 20 to 24. Next, we determined the number of eYLL by multiplying the average LE in a specific sex and age group by the excess deaths observed within that group. The following formula was applied:


$$eYLL_{c,s.t}=\underset{a}{\sum }eD_{a,c,s,t}\times LE_{a,c,s}^{2019}$$

where a—5-year age group; c—country; s—sex; t—year (2020–2023); eD_a,c,s,t_—country-, age-group- and sex-specific excess deaths in a year t; $LE_{a,c,s}^{2019}$—country-, age-group- and sex-specific remaining life expectancy in 2019.

To quantify eYPPLL, we first derived sex-specific work-period length for each country (WP_c,s,_). This was done by subtracting the sex- and country-specific average age of starting a first regular job (EntryAge_c,s_) (38) from the sex- and country-specific effective age of labour market exit (ExitAge_c,s_) (39).


$$WP_{c,s}=ExitAge_{c,s}-EntryAge_{c,s}$$

EntryAge_c,s_ was derived from the EU Statistics on Income and Living Conditions (EU-SILC) survey conducted among member states’ populations (survey item PL190: “When began first regular job”). For ExitAge_c,s_, we used European Commission data on the Cohort Simulation Model’s cumulated exit probabilities for the reference age group 51–74 for the year 2023 [Tables II.1.46 & II.1.47 in (39)].

Next, for each country c, sex s, and calendar year t, we calculated eYPPLL assuming that a death at a particular age group occurs at the mid-point age (MP_a,c,s_), e.g., at the age of 32 for the group 30–34, and summed years lost across the working age (aged 20–64) population (age groups <20 are excluded by definition). Therefore, the following formula was applied:


$$eYPPLL_{c,s.t}=\underset{a=20-64}{\sum }eD_{a,c,s,t}\times {\left(WP_{c,s}-MP_{a,c,s}\right)}^{+}$$

where (x)^+^ = max(0,x) ensures that deaths occurring at or above the retirement age add zero productive years lost.

### 2.6 Sensitivity analysis

We evaluated the robustness of excess mortality estimates to alternative trend specifications (S1-S4; deviations of the final specification from the remaining three) and alternative forecasting approaches. For the latter, we applied two commonly used time-series models, Autoregressive Integrated Moving Average (ARIMA) and exponential smoothing (ETS) (40) and compared their forecasts with those from the final OLS specification.

For ARIMA and ETS, we selected the most appropriate specification based on systematic model searches using AIC. Specifically, for the ARIMA model we evaluated all combinations of ARIMA(p,d,q) with *p* = 0,1,2, d = 0,1 and q = 0,1,2, and chose the model with the lowest AIC. For the ETS, we considered variants without trend, with additive and multiplicative trend components, and with the damping parameter controlling trend flattening (40). Again, the ETS model with the lowest AIC was chosen.

For eYLL, we examined robustness to alternative life expectancy assumption—projected LE in 2050 (data from the United Nations World Population Prospects 2024) (37) following Martinez et al. (27). For eYPPLL, we examined robustness to alternative definitions of productive lifespan by replacing country- and sex-specific labour-market entry and exit ages with a fixed working life span from ages 20 to 64.

### 2.7 Software

All analyses, data pre-processing and visualisations were carried out in R (v. 4.4.1) using RStudio (v. 2024.09) and Python (3.11.6) with libraries pandas (2.2.3), numpy (2.2.1) and statsmodels (0.14.4).

## 3 Results

### 3.1 Evaluation of baseline trend specifications

To identify the forecasting model to be applied uniformly across all countries, sexes, and age groups, we compared the performance of the four candidate specifications (S1–S4) using three goodness-of-fit metrics (AIC, MAE and RMSE) computed on pre-pandemic data. In total, 4,032 models (28 countries × 2 sexes × 18 age groups × 4 specifications) were evaluated.

Across all comparisons, the short-baseline linear model (S2) achieved the best performance. For AIC, S2 obtained 3,057 wins, followed by S4 (975), while S1 and S3 had no wins. For MAE, S2 achieved 1,332 wins, again outperforming S4 (920), S1 (71) and S3 (55). For RMSE, S2 achieved 1,290 wins, followed by S4 (865), with S3 (36) and S1 (28) far behind. Summed across all three metrics, S2 received 5,679 wins, more than double the next-best specification (S4: 2760 wins). Based on these results, S2 was selected as the final forecasting model. This specification corresponds to a linear trend fitted over the 2010–2019 baseline window multiverse, combined with outlier trimming and averaging as described in Section 2.4. This model, therefore, served as the basis for generating expected mortality for 2020–2023 in all subsequent analyses.

### 3.2 Excess deaths and excess mortality rates

We identified a total of 1,540,034 excess deaths (eD; Table 1) in the 28 European countries across 2020–2023. This burden was unequally distributed over time, with the highest figure estimated for 2021 (562,798 eD), followed by 2020 (451,869), 2022 (421,595), and 2023 (103,771). The highest number of eD across the whole period was identified in Central and Eastern Europe (CEE; 524,399; 34.1% of all). In Southern Europe (SE), we identified 506,309 eD (32.9%), in Western Europe (WE)—425,769 eD (27.6%) and in Northern Europe (NE)—83,557 eD (5.4%). The countries with the highest burden of eD were Italy (266,462; 17.3% of all), Poland (213,041; 13.8%), Germany (164,382; 10.7%), France (156,840; 10.2%) and Spain (136,756; 8.9%). Men accounted for 54.8% (844,449) of all eD, but this share varied across countries, with less than half of male eD in Cyprus (42.7%), Ireland (44.1%), Lithuania (43.7%), Malta (46.2%), and Portugal (41.9%); and >60% in Germany (63.9%), Greece (61.4%), Norway (60.6%) and Sweden (66.2%).

**Table 1:** Excess deaths in 28 European countries in 2020–2023.

Country	2020		2021		2022		2023		Total
	Males	Females	Males	Females	Males	Females	Males	Females	
**CEE**	**82,451 (68,001; 96,902)**	**61,296 (48,621; 73,970)**	**157,689 (140,700; 174,679)**	**139,615 (124,685; 154,546)**	**49,756 (29,885; 69,627)**	**45,769 (27,996; 63,541)**	**−5,999 (−29,473; 17,476)**	**−6,178 (−27,272; 14,915)**	**524,399 (383,143; 665,655)**
Bulgaria	9,744 (9,038; 10,451)	6,571 (5,322; 7,820)	21,606 (20,653; 22,559)	19,632 (18,145; 21,120)	5,782 (4,562; 7,002)	4,319 (2,584; 6,053)	−2,953 (−4,447; −1,459)	−3,766 (−5,772; −1,759)	60,936 (50,085; 71,787)
Czechia	8,710 (6,714; 10,706)	7,086 (5,518; 8,654)	16,383 (14,143; 18,623)	11,322 (9,451; 13,194)	4,596 (2,038; 7,154)	3,961 (1,698; 6,225)	606 (−2,347; 3,558)	−661 (−3,387; 2,064)	52,003 (33,828; 70,178)
Hungary	4,921 (3,301; 6,541)	4,999 (3,689; 6,309)	13,455 (11,435; 15,475)	11,151 (9,594; 12,708)	3,498 (1,014; 5,983)	2,509 (650; 4,368)	−93 (−3,106; 2,920)	−1,331 (−3,495; 834)	39,109 (23,081; 55,137)
Poland	35,548 (29,864; 41,233)	26,495 (22,065; 30,925)	58,024 (51,380; 64,667)	51,960 (46,600; 57,321)	20,376 (12,592; 28,160)	20,578 (13,917; 27,239)	−690 (−9,886; 8,507)	748 (−7,262; 8,759)	213,041 (159,269; 266,812)
Romania	20,928 (17,632; 24,223)	13,851 (10,650; 17,052)	38,220 (34,393; 42,047)	36,396 (32,807; 39,985)	12,219 (7,909; 16,528)	11,014 (6,995; 15,033)	−3,291 (−8,325; 1,743)	−1,952 (−6,661; 2,757)	127,384 (95,400; 159,368)
Slovakia	2,600 (1,452; 3,748)	2,293 (1,377; 3,210)	10,002 (8,695; 11,308)	9,154 (8,090; 10,219)	3,285 (1,770; 4,799)	3,388 (2,154; 4,623)	422 (−1,363; 2,207)	783 (−695; 2,260)	31,927 (21,480; 42,374)
**NE**	**8,245 (656; 15,833)**	**5,702 (724; 10,680)**	**13,274 (4,310; 22,237)**	**13,179 (6,883; 19,474)**	**14,757 (4,077; 25,437)**	**15,265 (7,207; 23,322)**	**7,976 (−4,712; 20,664)**	**5,160 (−4,866; 15,186)**	**83,557 (14,280; 152,834)**
Denmark	−61 (−1,183; 1,062)	−312 (−1,324; 701)	644 (−770; 2,059)	685 (−536; 1,906)	1,329 (−447; 3,105)	1,494 (1; 2,988)	605 (−1,575; 2,785)	310 (−1,487; 2,107)	4,695 (−7,321; 16,711)
Estonia	161 (−205; 527)	125 (−175; 424)	1,524 (1,088; 1,960)	1,551 (1,183; 1,919)	1,010 (487; 1,533)	912 (463; 1,360)	475 (−151; 1,101)	88 (−455; 632)	5,846 (2,235; 9,456)
Finland	473 (−613; 1,560)	240 (−345; 825)	1,377 (143; 2,612)	1,041 (325; 1,758)	3,603 (2,161; 5,045)	3,756 (2,854; 4,658)	3,025 (1,354; 4,695)	2,546 (1,443; 3,650)	16,063 (7,322; 24,803)
Ireland	172 (−617; 961)	305 (−288; 897)	1,076 (168; 1,985)	897 (204; 1,589)	896 (−179; 1,971)	1,422 (580; 2,265)	828 (−446; 2,103)	1,138 (102; 2,174)	6,735 (−475; 13,946)
Latvia	419 (−30; 868)	542 (113; 972)	3,269 (2,754; 3,784)	3,663 (3,122; 4,204)	1,881 (1,272; 2,491)	1,679 (1,009; 2,350)	870 (142; 1,598)	141 (−681; 964)	12,466 (7,701; 17,231)
Lithuania	2,889 (1,707; 4,071)	2,272 (1,576; 2,967)	3,706 (2,278; 5,133)	5,102 (4,226; 5,977)	1,653 (0; 3,306)	2,941 (1,786; 4,097)	−622 (−2,558; 1,314)	−486 (−1,932; 961)	17,455 (7,084; 27,826)
Sweden	4,223 (2,694; 5,751)	2,859 (1,948; 3,771)	1,330 (−471; 3,131)	−185 (−1,488; 1,118)	1,916 (−276; 4,109)	1,239 (−578; 3,055)	1,418 (−1,248; 4,084)	634 (−1,750; 3,019)	13,435 (−1,169; 28,039)
Norway	−33 (−1,097; 1,032)	−329 (−781; 123)	347 (−880; 1,574)	425 (−153; 1,004)	2,468 (1,058; 3,877)	1,821 (1,092; 2,549)	1,377 (−230; 2,984)	787 (−105; 1,679)	6,863 (−1,096; 14,821)
**SE**	**96,568 (75,075; 118,061)**	**91,486 (75,864; 107,107)**	**71,990 (47,240; 96,740)**	**56,516 (37,262; 75,771)**	**69,511 (40,701; 98,321)**	**73,778 (49,778; 97,777)**	**24,851 (−8,770; 58,471)**	**21,610 (−7,553; 50,772)**	**506,309 (309,597; 703,020)**
Croatia	2,448 (1,616; 3,279)	2,323 (1,625; 3,021)	5,702 (4,757; 6,648)	5,361 (4,529; 6,193)	2,852 (1,745; 3,960)	3,257 (2,226; 4,288)	728 (−577; 2,033)	131 (−1,128; 1,390)	22,802 (14,793; 30,812)
Cyprus	1 (−153; 154)	30 (−127; 188)	351 (106; 595)	300 (76; 524)	247 (−92; 586)	363 (66; 660)	−109 (−553; 334)	−38 (−413; 337)	1,145 (−1,090; 3,380)
Greece	2,951 (1,076; 4,826)	2,681 (888; 4,474)	10,040 (7,709; 12,372)	7,858 (5,454; 10,262)	9,304 (6,633; 11,975)	6,243 (3,234; 9,253)	3,808 (687; 6,929)	−386 (−4,068; 3,296)	42,500 (21,614; 63,386)
Italy	51,458 (41,016; 61,900)	45,470 (39,528; 51,413)	33,682 (22,094; 45,270)	27,719 (20,449; 34,989)	35,883 (22,682; 49,084)	39,423 (30,501; 48,346)	16,603 (1,587; 31,619)	16,224 (5,554; 26,894)	266,462 (183,410; 349,514)
Malta	166 (58; 274)	97 (−2; 196)	93 (−64; 251)	178 (45; 310)	141 (−72; 354)	123 (−40; 286)	24 (−252; 299)	95 (−113; 303)	917 (−439; 2,272)
Portugal	4,046 (2,113; 5,980)	5,151 (3,428; 6,873)	4,659 (2,422; 6,896)	4,937 (2,948; 6,926)	3,019 (391; 5,647)	5,179 (2,872; 7,485)	311 (−2,798; 3,421)	1,415 (−1,224; 4,055)	28,717 (10,151; 47,284)
Slovenia	1,385 (935; 1,834)	1,708 (1,416; 1,999)	1,387 (822; 1,952)	896 (521; 1,272)	616 (−74; 1,307)	757 (291; 1,224)	313 (−521; 1,148)	−54 (−635; 527)	7,009 (2,755; 11,263)
Spain	34,113 (28,413; 39,814)	34,025 (29,107; 38,943)	16,075 (9,394; 22,757)	9,267 (3,240; 15,295)	17,449 (9,489; 25,408)	18,432 (10,629; 26,235)	3,173 (−6,342; 12,688)	4,222 (−5,526; 13,970)	136,756 (78,404; 195,108)
**WE**	**61,780 (35,981; 87,578)**	**44,343 (26,375; 62,310)**	**72,774 (43,562; 101,985)**	**37,762 (14,905; 60,619)**	**82,357 (48,485; 116,230)**	**70,404 (41,880; 98,928)**	**36,470 (−2,758; 75,699)**	**19,880 (−14,662; 54,422)**	**425,769 (193,768; 657,770)**
Austria	3,821 (2,471; 5,171)	2,994 (2,079; 3,909)	4,150 (2,560; 5,741)	2,543 (1,265; 3,821)	4,227 (2,326; 6,128)	3,324 (1,786; 4,862)	2,586 (345; 4,827)	1,024 (−793; 2,841)	24,670 (12,040; 37,301)
Belgium	8,402 (6,180; 10,625)	8,614 (7,282; 9,946)	3,637 (1,244; 6,030)	379 (−1,119; 1,876)	3,867 (1,202; 6,533)	4,226 (2,475; 5,976)	1,623 (−1,379; 4,626)	1,576 (−473; 3,625)	32,324 (15,411; 49,237)
France	26,884 (18,911; 34,857)	21,531 (17,878; 25,184)	25,681 (16,928; 34,433)	15,852 (11,248; 20,457)	27,048 (17,195; 36,900)	25,100 (19,444; 30,755)	8,156 (−3,014; 19,326)	6,588 (−178; 13,354)	156,840 (98,413; 215,267)
Germany	14,442 (2,896; 25,989)	6,263 (−4,172; 16,698)	30,269 (16,907; 43,630)	13,645 (422; 26,868)	41,159 (25,430; 56,889)	32,439 (15,972; 48,906)	19,121 (699; 37,544)	7,043 (−13,001; 27,087)	164,382 (45,154; 283,611)
Luxembourg	128 (15; 242)	88 (12; 164)	13 (−165; 191)	32 (−83; 147)	−82 (−332; 169)	14 (−144; 172)	−213 (−542; 117)	23 (−181; 228)	4 (−1,422; 1,430)
Netherlands	8,101 (5,509; 10,694)	4,853 (3,296; 6,410)	9,024 (6,088; 11,960)	5,310 (3,172; 7,449)	6,138 (2,664; 9,612)	5,301 (2,347; 8,256)	5,196 (1,133; 9,258)	3,625 (−36; 7,286)	47,548 (24,172; 70,924)
**Total**	**249,043 (179,713; 318,374)**	**202,826 (151,584; 254,068)**	**315,726 (235,811; 395,642)**	**247,072 (183,735; 310,409)**	**216,381 (123,148; 309,614)**	**205,214 (126,861; 283,568)**	**63,299 (−45,712; 172,310)**	**40,472 (−54,352; 135,295)**	**1,540,034 (900,788; 2,179,279)**

Each cell contains a point estimate and a 95% confidence interval in brackets. CEE, Central and Eastern Europe; NE, Northern Europe; SE, Southern Europe; WE, Western Europe. Bold font represents total values for countries within each of four European regions.

The age-standardised excess mortality rate per 100,000 population (eM_R) across all countries and the whole period was 87 (CI: 84–91); for men, it was 127 (CI: 123–132) and for women, 61 (CI: 58–63). It was 2021 when eM_Rs were the highest (126; CI: 119–133), followed by 2020 (86; CI: 80–91), 2022 (85; CI: 77–93), and 2023 (21; CI: 12–30). For all years, the eM_Rs were higher among men than women (Supplementary Table S1).

CEE experienced the highest excess mortality; for all years combined, eM_R was 190 (CI: 182–198) there, and it was more than double the level of SE states (84; CI: 78–91), and more than triple of rates in WE (58; CI: 52–64) and NE (54; CI: 47–62) countries (Supplementary Table S1). As for the time distribution of eM_R, CEE countries experienced the highest burden in 2020–2022, but in 2023, these countries had negative eM_Rs. For individual countries, the highest four-year eM_R was observed in Bulgaria (257; CI: 239–274), Romania (209; CI: 188–230), and Slovakia (201; CI: 173–229), and the lowest in Denmark (13; CI: −10–37), Luxembourg (24; CI: −3–50), and Norway (26; CI: 7–45). For sex-specific eM_Rs, the country ranking was not much different, with CEE and three Baltic states (Lithuania, Latvia, and Estonia) having the highest eM_Rs, and Northern and Western countries experiencing the lowest values of this measure (Supplementary Table S1).

**Figure 1:**
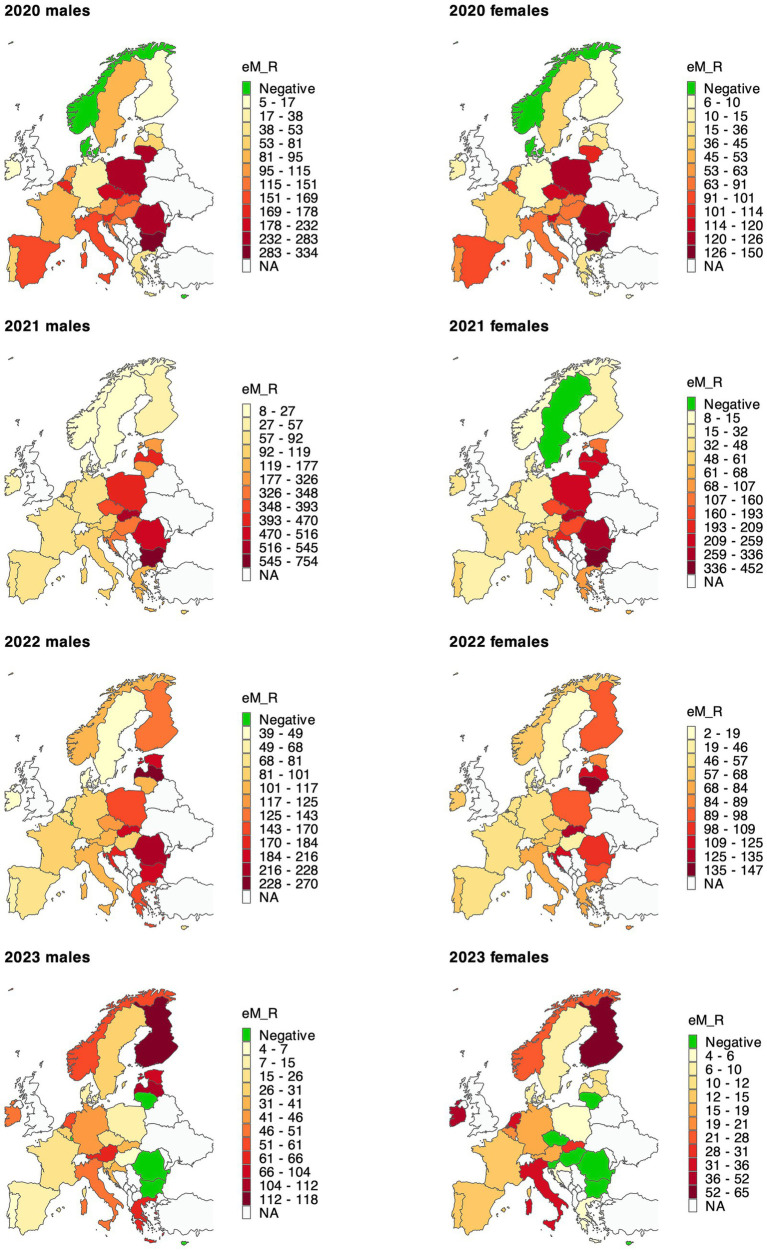
Age-standardised excess mortality rate per 100,000 population in 28 countries in 2020–2023. Detailed results with confidence intervals are available in Supplementary Table S1. EM_R—age-standardised excess mortality rate per 100,000 population. Each map uses its own colour scale because the magnitude of excess mortality varies substantially across years (e.g., 2021 shows much higher values than 2023) and sexes.

For particular years, 2021 shows the greatest eM_Rs, with Bulgaria, Slovakia, and Romania exhibiting the highest values in both sexes (Figure 1; detailed data in Supplementary Table S1). However, for some countries, we observed eM_Rs to be higher in 2022 than in 2021, and this was true for Austria, Belgium, Denmark, Finland, France, Germany, Italy, Norway, and Spain, among others. In 2023, some of the countries that were most burdened in previous years had shown reduced (negative excess) mortality, and these were Bulgaria, Lithuania, Hungary and Romania. Additionally, Greece (men), as well as Finland and the Netherlands (both sexes), all of which were mildly affected in previous years, experienced a relatively high excess mortality burden in 2023.

### 3.3 Excess years of life lost

1,540,034 excess deaths translated to a total of 16,695,365 excess years of life lost (eYLL; Table 2). Time distribution of eYLL reflects excess death patterns, with the highest share of eYLL in 2021 (41.0%) and the lowest in 2023 (7.7%). Analogously to eD, the region most burdened in terms of eYLL was CEE, with 5.8 million eYLL (34.6% of total); a large share of the total eYLL in this region resulted from higher excess mortality in younger age groups. For Southern and Western countries, the respective eYLL (% of total) were 5.1 million (30.8%) and 4.8 million (28.5%). The countries with the highest eYLL were Italy (2.7 million; 16.1%), Poland (2.4 million; 14.4% of all), Germany (2.0 million; 12.0%), France (1.6 million; 9.7%), and Spain (1.5 million; 8.9%). Men accounted for 58.0% (9.7 million) of all eYLL, with the highest shares for this measure identified in Sweden (76.8%), Austria (65.7%) and Germany (64.4%).

**Table 2:** Excess years of life lost (eYLL) in 28 European countries in 2020–2023.

Country	2020		2021		2022		2023		Total
	Males	Females	Males	Females	Males	Females	Males	Females	
**CEE**	**911,235 (673,217; 1,149,253)**	**641,146 (476,576; 805,717)**	**1,941,524 (1,660,305; 2,222,744)**	**1,642,874 (1,446,125; 1,839,622)**	**516,908 (185,616; 848,200)**	**390,851 (154,067; 627,636)**	**−154,047 (−543,296; 235,202)**	**−115,478 (−395,276; 164,319)**	**5,775,013 (3,657,334; 7,892,692)**
Bulgaria	107,311 (95,426; 119,195)	79,594 (65,220; 93,968)	244,134 (228,161; 260,107)	233,996 (215,537; 252,456)	51,099 (30,560; 71,637)	49,788 (27,364; 72,213)	−31,066 (−56,456; −5,676)	−30,083 (−56,767; −3,399)	704,773 (549,045; 860,500)
Czechia	81,400 (51,022; 111,778)	56,619 (35,187; 78,051)	196,392 (161,628; 231,156)	132,134 (106,605; 157,664)	48,871 (8,329; 89,413)	34,772 (3,883; 65,662)	4,031 (−43,315; 51,376)	−12,580 (−49,924; 24,764)	541,638 (273,413; 809,863)
Hungary	56,445 (24,131; 88,759)	55,624 (35,093; 76,156)	189,094 (150,156; 228,032)	159,940 (136,022; 183,858)	46,773 (364; 93,182)	38,376 (10,003; 66,750)	−3,706 (−58,603; 51,192)	−930 (−33,930; 32,069)	541,616 (263,235; 819,998)
Poland	417,993 (321,630; 514,357)	268,902 (208,164; 329,640)	744,081 (632,683; 855,479)	591,318 (518,570; 664,066)	248,994 (119,423; 378,564)	191,660 (102,892; 280,428)	−38,984 (−190,534; 112,567)	−12,256 (−117,029; 92,517)	2,411,708 (1,595,799; 3,227,617)
Romania	221,244 (171,853; 270,634)	158,240 (122,330; 194,151)	433,656 (374,087; 493,225)	415,851 (373,918; 457,784)	81,202 (11,076; 151,328)	51,434 (2,232; 100,637)	−88,103 (−169,975; −6,231)	−61,036 (−118,368; −3,704)	1,212,488 (767,152; 1,657,824)
Slovakia	26,842 (9,155; 44,530)	22,166 (10,582; 33,751)	134,168 (113,591; 154,745)	109,635 (95,475; 123,795)	39,970 (15,864; 64,076)	24,820 (7,694; 41,947)	3,781 (−24,413; 31,975)	1,407 (−19,257; 22,072)	362,790 (208,690; 516,890)
**NE**	**101,589 (−24,852; 228,030)**	**49,915 (−28,957; 128,787)**	**184,058 (35,192; 332,925)**	**145,073 (45,325; 244,820)**	**187,427 (11,208; 363,646)**	**149,418 (23,193; 275,643)**	**130,408 (−77,814; 338,631)**	**66,456 (−89,708; 222,619)**	**1,014,344 (−106,412; 2,135,100)**
Denmark	−2,009 (−19,818; 15,801)	−437 (−15,933; 15,060)	2,675 (−19,252; 24,602)	10,311 (−8,453; 29,075)	12,192 (−14,495; 38,880)	18,828 (−4,127; 41,784)	7,503 (−24,404; 39,409)	6,987 (−20,630; 34,605)	56,052 (−127,113; 239,217)
Estonia	3,308 (−3,781; 10,398)	1,324 (−3,374; 6,023)	20,332 (12,080; 28,583)	15,517 (9,333; 21,701)	15,439 (5,683; 25,196)	8,138 (259; 16,016)	9,711 (−1,864; 21,287)	2,393 (−7,487; 12,272)	76,163 (10,848; 141,477)
Finland	12,366 (−5,997; 30,729)	353 (−8,599; 9,305)	15,502 (−6,086; 37,089)	8,010 (−3,330; 19,350)	36,350 (10,855; 61,846)	32,626 (18,152; 47,100)	32,568 (2,711; 62,425)	23,156 (5,292; 41,019)	160,931 (12,999; 308,863)
Ireland	5,133 (−7,781; 18,048)	6,262 (−2,822; 15,346)	18,550 (3,467; 33,633)	13,513 (1,789; 25,236)	15,363 (−2,644; 33,369)	17,475 (2,506; 32,445)	12,257 (−9,429; 33,944)	11,221 (−7,891; 30,333)	99,775 (−22,805; 222,355)
Latvia	3,520 (−4,386; 11,427)	2,707 (−3,673; 9,087)	44,265 (34,742; 53,789)	37,437 (29,000; 45,874)	29,279 (17,595; 40,962)	15,545 (4,852; 26,239)	17,623 (3,376; 31,871)	1,096 (−12,162; 14,354)	151,472 (69,342; 233,603)
Lithuania	39,761 (17,494; 62,028)	25,161 (13,916; 36,406)	59,660 (34,367; 84,952)	57,689 (44,234; 71,144)	30,956 (2,200; 59,711)	31,637 (15,059; 48,215)	10,053 (−23,220; 43,327)	1,465 (−18,508; 21,438)	256,382 (85,542; 427,221)
Sweden	40,278 (16,983; 63,574)	17,018 (2,798; 31,239)	21,827 (−5,399; 49,052)	−604 (−19,452; 18,245)	22,912 (−9,477; 55,301)	8,245 (−16,729; 33,220)	20,431 (−18,090; 58,951)	7,254 (−24,515; 39,023)	137,362 (−73,881; 348,605)
Norway	−770 (−17,565; 16,025)	−2,474 (−11,269; 6,321)	1,248 (−18,728; 21,224)	3,200 (−7,796; 14,195)	24,936 (1,491; 48,380)	16,923 (3,221; 30,624)	20,261 (−6,893; 47,415)	12,884 (−3,807; 29,575)	76,207 (−61,345; 213,759)
**SE**	**967,482 (664,766; 1,270,198)**	**756,703 (562,021; 951,385)**	**884,969 (538,897; 1,231,041)**	**632,882 (399,884; 865,881)**	**702,554 (302,974; 1,102,134)**	**636,074 (355,762; 916,386)**	**311,372 (−151,167; 773,912)**	**250,435 (−85,075; 585,945)**	**5,142,471 (2,588,062; 7,696,881)**
Croatia	21,100 (10,029; 32,171)	21,180 (12,583; 29,777)	55,928 (42,574; 69,283)	54,019 (43,212; 64,826)	23,233 (6,771; 39,695)	23,344 (9,674; 37,014)	6,064 (−13,797; 25,925)	776 (−15,992; 17,545)	205,645 (95,055; 316,235)
Cyprus	364 (−2,201; 2,929)	788 (−1,465; 3,041)	5,073 (1,037; 9,109)	5,196 (1,905; 8,487)	1,884 (−3,713; 7,481)	3,697 (−786; 8,180)	−114 (−7,393; 7,165)	−268 (−5,955; 5,418)	16,620 (−18,570; 51,811)
Greece	26,309 (4,722; 47,896)	27,684 (7,822; 47,546)	119,995 (91,819; 148,170)	93,279 (67,509; 119,050)	63,227 (29,330; 97,124)	61,685 (30,335; 93,034)	7,967 (−32,409; 48,343)	10,905 (−27,088; 48,898)	411,050 (172,040; 650,061)
Italy	515,535 (379,669; 651,401)	359,428 (285,229; 433,627)	418,210 (269,636; 566,783)	299,649 (212,296; 387,003)	363,239 (195,867; 530,611)	322,070 (217,464; 426,675)	227,001 (38,442; 415,560)	178,243 (54,770; 301,717)	2,683,375 (1,653,373; 3,713,378)
Malta	2,381 (377; 4,385)	−361 (−2,267; 1,545)	1,638 (−1,439; 4,715)	848 (−2,081; 3,778)	2,744 (−1,377; 6,865)	−506 (−4,010; 2,997)	−67 (−5,802; 5,668)	−1,742 (−6,855; 3,371)	4,936 (−23,455; 33,326)
Portugal	45,358 (16,896; 73,821)	40,190 (18,005; 62,375)	52,703 (19,189; 86,216)	42,828 (16,141; 69,515)	34,734 (−4,836; 74,303)	37,808 (5,968; 69,648)	10,037 (−36,716; 56,790)	6,029 (−31,420; 43,479)	269,687 (3,227; 536,148)
Slovenia	11,165 (3,888; 18,443)	11,293 (6,954; 15,631)	16,272 (7,086; 25,457)	9,330 (3,594; 15,066)	7,053 (−4,088; 18,194)	6,917 (−223; 14,058)	3,226 (−10,072; 16,524)	−1,135 (−10,182; 7,912)	64,121 (−3,044; 131,286)
Spain	345,268 (251,386; 439,151)	296,502 (235,160; 357,844)	215,150 (108,994; 321,306)	127,732 (57,309; 198,155)	206,441 (85,019; 327,862)	181,059 (97,340; 264,779)	57,258 (−83,420; 197,935)	57,627 (−42,352; 157,605)	1,487,037 (709,437; 2,264,637)
**WE**	**580,079 (184,957; 975,200)**	**313,954 (75,270; 552,638)**	**940,423 (492,082; 1,388,763)**	**465,206 (171,042; 759,371)**	**962,596 (445,058; 1,480,133)**	**699,725 (339,770; 1,059,681)**	**515,623 (−81,551; 1,112,798)**	**285,931 (−147,082; 718,943)**	**4,763,537 (1,479,546; 8,047,527)**
Austria	36,171 (12,565; 59,776)	22,629 (8,679; 36,580)	57,023 (29,032; 85,015)	27,788 (9,490; 46,086)	48,392 (15,001; 81,784)	30,443 (7,759; 53,127)	34,240 (−5,309; 73,789)	11,121 (−16,467; 38,709)	267,808 (60,750; 474,866)
Belgium	79,530 (44,047; 115,014)	62,703 (43,498; 81,907)	52,332 (13,337; 91,326)	15,527 (−6,915; 37,969)	46,549 (2,845; 90,253)	37,198 (10,680; 63,716)	18,983 (−30,218; 68,184)	12,634 (−18,581; 43,849)	325,456 (58,693; 592,219)
France	244,891 (108,131; 381,651)	158,912 (102,682; 215,143)	311,061 (157,729; 464,394)	163,019 (91,894; 234,145)	317,805 (143,383; 492,227)	232,973 (145,313; 320,633)	120,225 (−77,950; 318,400)	65,269 (−39,838; 170,377)	1,614,157 (631,344; 2,596,970)
Germany	136,447 (−23,263; 296,156)	28,755 (−96,342; 153,852)	414,719 (232,389; 597,049)	190,361 (39,031; 341,691)	471,077 (259,526; 682,628)	340,249 (156,994; 523,504)	272,903 (26,900; 518,905)	155,998 (−64,131; 376,127)	2,010,509 (531,105; 3,489,913)
Luxembourg	383 (−1,939; 2,705)	734 (−880; 2,348)	−1,117 (−4,888; 2,654)	568 (−1,977; 3,113)	−1,260 (−6,496; 3,976)	−661 (−4,271; 2,949)	−4,158 (−11,016; 2,701)	215 (−4,512; 4,942)	−5,296 (−35,979; 25,388)
Netherlands	82,657 (45,416; 119,898)	40,220 (17,633; 62,807)	106,404 (64,483; 148,325)	67,943 (39,519; 96,367)	80,033 (30,799; 129,267)	59,522 (23,293; 95,751)	73,430 (16,042; 130,818)	40,694 (−3,553; 84,940)	550,903 (233,633; 868,173)
**Total**	**2,560,384 (1,498,088; 3,622,680)**	**1,761,718 (1,084,910; 2,438,526)**	**3,950,974 (2,726,476; 5,175,472)**	**2,886,035 (2,062,376; 3,709,694)**	**2,369,484 (944,856; 3,794,113)**	**1,876,068 (872,791; 2,879,345)**	**803,357 (−853,828; 2,460,542)**	**487,343 (−717,140; 1,691,827)**	**16,695,365 (7,618,529; 25,772,200)**

Each cell contains a point estimate and a 95% confidence interval in brackets. CEE, Central and Eastern Europe; NE, Northern Europe; SE, Southern Europe; WE, Western Europe. Bold font represents total values for countries within each of four European regions.

The age-standardised excess years of life lost per 100,000 population (eYLL_R) for all years and countries combined was 974 (CI: 922–1,026) and was higher in men (1,304; CI: 1,239–1,369) than in women (695; CI: 657–734; Supplementary Table S2). Clearly, time variation in this measure reflects what we observed in excess mortality, with 2021 being the most burdening year (for both sexes combined: eYLL_R = 1,581; CI: 1,482–1,681), and 2023 with the lowest rate of years lost (254; CI: 114–393). For all years, the eYLL_R was higher among men than women (Supplementary Table S2). In 2021, the eYLL_R was as much as 4,405 (CI: 4,176-4,634) in CEE (5,638; CI: 5,362–5,913 in men and 3,456; CI: 3,279–3,634 in women) and only 782 (CI: 549–1,015) in NE. In 2023, the measure was negative in CEE (Supplementary Table S2).

**Figure 2:**
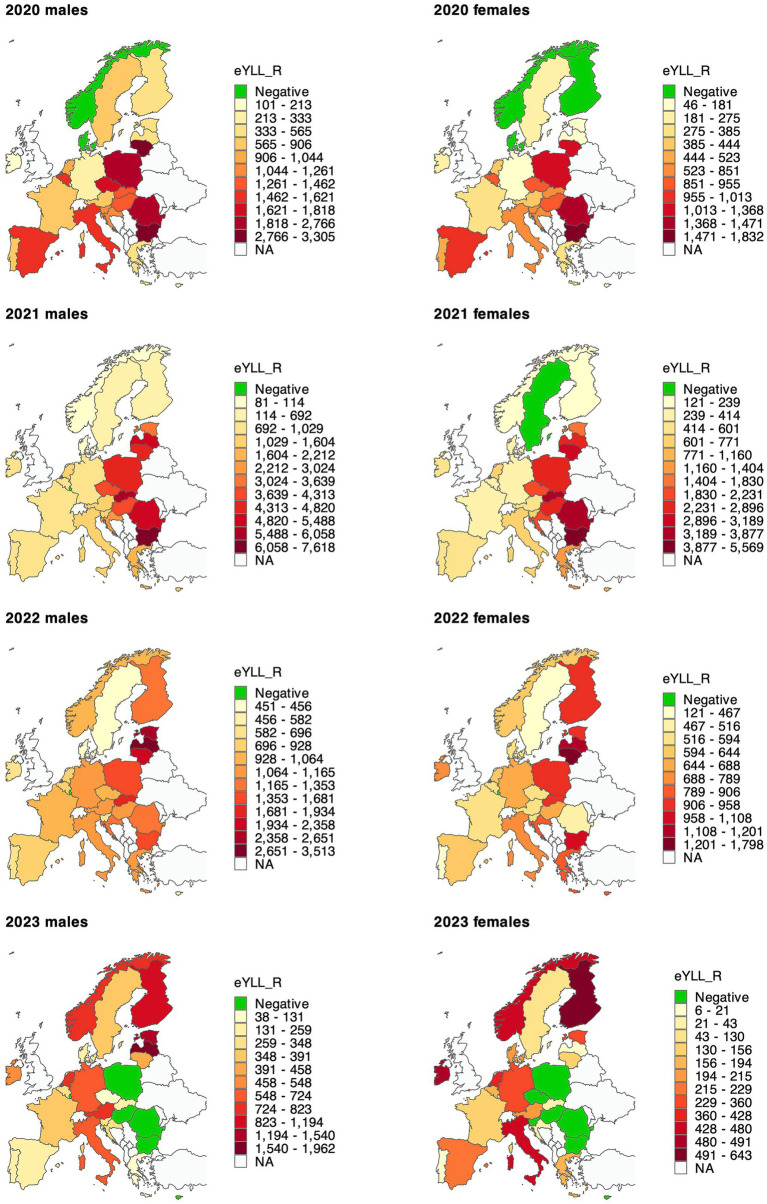
Age-standardised excess years of life lost per 100,000 population in 28 countries in 2020–2023. Detailed results with confidence intervals are available in Supplementary Table S2. eYLL_R—age-standardised excess years of life lost rate per 100,000 population. Each map uses its own colour scale because the magnitude of excess years of life lost varies substantially across years (e.g., 2021 shows much higher values than 2023) and sexes.

For individual countries, the highest four-year eYLL_R was observed in Bulgaria (2,930; CI: 2,672–3,188), Lithuania (2,394; CI: 1,699–3,089) and Slovakia (2,055; CI: 1,695–2,414), and the lowest in Luxembourg (56; CI: −447–558), Denmark (168; CI: −192-527), and Norway (255; CI: −58–568). For sex-specific eYLL_Rs, Bulgaria experienced the highest four-year burden in both men (3,720; CI: 3,456-3,983) and women (2,366; CI: 2,125–2,607). Most of the highest year-specific eYLL_Rs were observed in 2021 and all of these referred to CEE or three Baltic states (Latvia, Lithuania, Estonia); yet, these countries contributed with the highest eYLL_Rs also in other years, e.g., Bulgaria, Lithuania, Poland and Romania in 2020; and Estonia, Lithuania, Latvia and Slovakia in 2022 (Figure 2; Supplementary Table S2). Most of these countries had lower or even negative rates of years lost in 2023, but Estonia and Latvia remained the ones with the high burden also in the last year. Interestingly, for women in Sweden, eYLL_R was higher in 2023 than in 2022, exhibiting a delayed effect of the pandemic on years of life lost. In contrast, for the last year analysed, we note significantly decreased eYLL_R compared to a hypothetical non-pandemic scenario in Bulgaria and Romania (both sexes), Malta and Czechia (women), and Luxembourg (men).

### 3.4 Excess years of potential productive life lost

Among the working-age Europeans (20–64 years old), excess mortality resulted in 1,997,095 eYPPLL (Table 3), 12.0% of total eYLL, highlighting the predominant impact of the pandemic on mortality among those aged 65+. As for the time distribution of eYPPLL, we observe a higher share of the burden in 2023 (10.8% of total) than in eYLL (7.7%) and excess deaths (6.7%). Therefore, for the last year analysed, we identified relatively more deaths among the working-age Europeans. As much as 41.0% of the total eYPPLL were identified in CEE, highlighting high mortality in the young-aged, while this share was 27.6% in WE and 22.7% in SE. The countries experiencing the highest eYPPLL were Poland (376,571 years lost; 18.9% of total), Germany (285,718; 14.3%), Italy (193,036; 9.7%), Spain (163,827; 8.2%), and France (144,116; 7.2%). Males accounted for 75.6% of total eYPPLL (1,509,928 years lost) in all countries combined, with the highest shares identified in Portugal (97.3%) and Estonia (90.8%). For several countries (e.g., Finland, Sweden, Austria), we identified negative eYPPLL among females in all years combined; therefore, the respective males’ share of >100% cannot be interpreted analogously to countries experiencing positive values for both sexes.

**Table 3:** Excess years of potential productive life lost (eYPPLL) in 28 European countries in 2020–2023.

Country	2020		2021		2022		2023		Total
	Males	Females	Males	Females	Males	Females	Males	Females	
**CEE**	**139,044 (74,327; 203,760)**	**49,468 (29,442; 69,493)**	**363,232 (286,328; 440,136)**	**149,174 (124,120; 174,228)**	**104,687 (14,540; 194,835)**	**37,606 (6,710; 68,501)**	**−22,484 (−128,235; 83,267)**	**−2,439 (−39,590; 34,712)**	**818,288 (367,643; 1,268,933)**
Bulgaria	19,225 (16,460; 21,991)	7,307 (6,128; 8,485)	46,234 (42,135; 50,333)	23,089 (21,126; 25,051)	3,272 (−2,249; 8,793)	3,936 (1,204; 6,668)	−9,020 (−16,070; −1,971)	−3,469 (−7,012; 74)	90,573 (61,722; 119,423)
Czechia	5,581 (89; 11,073)	3,034 (1,083; 4,985)	23,463 (16,997; 29,929)	10,432 (7,874; 12,990)	10,949 (3,074; 18,824)	2,627 (−628; 5,881)	7,089 (−2,352; 16,530)	−1,312 (−5,441; 2,817)	61,863 (20,697; 103,028)
Hungary	9,519 (−2,977; 22,016)	5,084 (477; 9,690)	45,057 (30,591; 59,523)	22,063 (16,687; 27,439)	12,473 (−3,934; 28,880)	5,110 (−1,169; 11,388)	2,063 (−16,494; 20,621)	3,372 (−3,913; 10,657)	104,742 (19,268; 190,215)
Poland	73,614 (44,743; 102,485)	18,679 (12,042; 25,315)	150,640 (118,119; 183,161)	45,915 (38,140; 53,690)	62,226 (25,321; 99,130)	22,870 (13,691; 32,049)	−4,435 (−47,157; 38,286)	7,063 (−3,612; 17,739)	376,571 (201,288; 551,855)
Romania	29,662 (18,505; 40,820)	15,382 (11,086; 19,677)	75,571 (60,939; 90,202)	40,303 (34,805; 45,801)	7,889 (−9,922; 25,699)	2,745 (−4,227; 9,718)	−20,839 (−42,203; 525)	−7,490 (−15,910; 929)	143,222 (53,072; 233,371)
Slovakia	1,442 (−2,493; 5,376)	−17 (−1,374; 1,340)	22,267 (17,547; 26,987)	7,374 (5,489; 9,258)	7,879 (2,249; 13,509)	318 (−2,161; 2,797)	2,658 (−3,959; 9,276)	−603 (−3,702; 2,496)	41,318 (11,596; 71,040)
**NE**	**20,678 (−11,376; 52,731)**	**2,593 (−10,637; 15,824)**	**41,306 (3,336; 79,275)**	**14,555 (−2,985; 32,096)**	**40,438 (−4,677; 85,553)**	**9,841 (−12,683; 32,365)**	**37,204 (−16,344; 90,752)**	**6,005 (−22,195; 34,206)**	**172,620 (−77,561; 422,802)**
Denmark	1,023 (−3,072; 5,117)	654 (−1,797; 3,105)	−87 (−4,881; 4,707)	1,223 (−1,895; 4,341)	3,328 (−2,355; 9,012)	1,006 (−2,900; 4,913)	2,558 (−4,103; 9,219)	759 (−4,044; 5,562)	10,464 (−25,048; 45,976)
Estonia	2,064 (−748; 4,877)	−215 (−926; 495)	4,948 (1,803; 8,093)	1,039 (−8; 2,086)	6,076 (2,459; 9,692)	644 (−748; 2,037)	5,777 (1,578; 9,976)	442 (−1,375; 2,260)	20,775 (2,034; 39,516)
Finland	4,060 (255; 7,865)	−626 (−1,843; 591)	129 (−4,729; 4,987)	−689 (−2,358; 981)	2,402 (−3,541; 8,345)	655 (−1,556; 2,867)	3,659 (−3,490; 10,808)	−200 (−2,997; 2,596)	9,390 (−20,260; 39,039)
Ireland	2,740 (−129; 5,609)	753 (−664; 2,171)	4,560 (1,278; 7,843)	1,311 (−896; 3,517)	3,031 (−838; 6,899)	1,299 (−1,780; 4,378)	3,985 (−600; 8,571)	−57 (−4,184; 4,071)	17,623 (−7,814; 43,059)
Latvia	−380 (−2,984; 2,224)	−545 (−1,551; 461)	13,296 (9,851; 16,741)	4,040 (2,587; 5,493)	11,564 (7,095; 16,034)	1,758 (−167; 3,683)	8,584 (2,947; 14,222)	−490 (−2,936; 1,955)	37,827 (14,842; 60,812)
Lithuania	10,784 (1,703; 19,865)	2,936 (675; 5,197)	18,134 (8,213; 28,056)	7,673 (5,093; 10,253)	9,504 (−1,521; 20,529)	5,237 (2,232; 8,242)	6,556 (−6,026; 19,137)	2,374 (−1,144; 5,891)	63,197 (9,224; 117,171)
Sweden	381 (−3,315; 4,078)	−968 (−3,336; 1,401)	1,842 (−2,834; 6,518)	−357 (−3,456; 2,742)	1,797 (−4,053; 7,648)	−2,675 (−6,604; 1,255)	1,936 (−5,280; 9,151)	276 (−4,551; 5,104)	2,233 (−33,430; 37,896)
Norway	6 (−3,085; 3,097)	604 (−1,194; 2,403)	−1,517 (−5,364; 2,330)	316 (−2,052; 2,684)	2,736 (−1,922; 7,394)	1,915 (−1,159; 4,989)	4,150 (−1,368; 9,669)	2,902 (−964; 6,767)	11,112 (−17,109; 39,333)
**SE**	**76,269 (19,775; 132,764)**	**31,022 (8,791; 53,253)**	**114,790 (49,666; 179,914)**	**44,940 (17,342; 72,538)**	**83,097 (8,089; 158,105)**	**32,016 (−1,675; 65,708)**	**57,410 (−28,765; 143,585)**	**14,785 (−25,871; 55,440)**	**454,329 (47,350; 861,307)**
Croatia	325 (−1,805; 2,455)	655 (−252; 1,563)	6,273 (3,618; 8,928)	4,004 (2,707; 5,301)	1,815 (−1,554; 5,184)	767 (−934; 2,468)	−420 (−4,549; 3,710)	343 (−1,769; 2,455)	13,763 (−4,538; 32,064)
Cyprus	387 (−188; 962)	425 (7; 843)	1,607 (669; 2,544)	832 (226; 1,438)	272 (−1,046; 1,589)	300 (−531; 1,131)	706 (−1,013; 2,425)	106 (−972; 1,183)	4,634 (−2,847; 12,115)
Greece	805 (−3,325; 4,934)	3,087 (1,231; 4,944)	18,807 (13,224; 24,390)	8,261 (5,885; 10,637)	3,692 (−3,252; 10,636)	2,578 (−391; 5,547)	−4,223 (−12,592; 4,146)	1,111 (−2,532; 4,754)	34,118 (−1,752; 69,988)
Italy	26,970 (9,197; 44,744)	9,884 (2,446; 17,323)	45,210 (25,947; 64,473)	18,015 (8,783; 27,247)	35,002 (13,634; 56,370)	13,472 (2,057; 24,886)	34,224 (10,488; 57,961)	10,258 (−3,592; 24,109)	193,036 (68,960; 317,112)
Malta	677 (203; 1,152)	−248 (−558; 62)	514 (−272; 1,299)	−395 (−880; 90)	860 (−268; 1,987)	−392 (−1,074; 289)	197 (−1,413; 1,806)	−605 (−1,517; 308)	608 (−5,779; 6,995)
Portugal	10,544 (3,233; 17,854)	1,024 (−1,324; 3,371)	9,028 (609; 17,446)	1,857 (−1,549; 5,263)	9,312 (−439; 19,063)	−163 (−4,708; 4,381)	6,966 (−4,361; 18,294)	−1,716 (−7,504; 4,072)	36,851 (−16,042; 89,745)
Slovenia	652 (−950; 2,254)	645 (39; 1,250)	1,800 (−329; 3,929)	613 (−273; 1,498)	1,142 (−1,496; 3,780)	1,315 (128; 2,503)	488 (−2,696; 3,672)	837 (−667; 2,342)	7,492 (−6,244; 21,228)
Spain	35,909 (13,409; 58,409)	15,549 (7,201; 23,897)	31,552 (6,199; 56,904)	11,753 (2,442; 21,063)	31,003 (2,510; 59,496)	14,140 (3,777; 24,504)	19,470 (−12,630; 51,570)	4,450 (−7,316; 16,217)	163,827 (15,592; 312,061)
**WE**	**55,815 (−23,004; 134,634)**	**3,465 (−28,039; 34,969)**	**139,954 (50,804; 229,105)**	**32,292 (−5,633; 70,216)**	**149,810 (48,671; 250,950)**	**44,673 (−1,071; 90,416)**	**108,678 (−6,596; 223,952)**	**17,172 (−37,620; 71,964)**	**551,858 (−2,489; 1,106,206)**
Austria	1,559 (−3,135; 6,253)	−770 (−2,435; 896)	9,339 (3,824; 14,855)	−166 (−2,642; 2,309)	7,059 (627; 13,491)	−29 (−3,351; 3,294)	5,641 (−1,940; 13,222)	28 (−4,232; 4,288)	22,662 (−13,284; 58,609)
Belgium	5,978 (−28; 11,984)	1,247 (−1,918; 4,412)	6,699 (−225; 13,623)	4,092 (355; 7,829)	7,307 (−601; 15,214)	3,166 (−1,222; 7,555)	3,610 (−5,372; 12,591)	2,471 (−2,717; 7,658)	34,570 (−11,727; 80,867)
France	15,257 (−12,160; 42,674)	1,801 (−6,117; 9,718)	35,866 (4,312; 67,420)	1,618 (−8,557; 11,793)	45,919 (9,728; 82,110)	9,389 (−3,368; 22,146)	32,848 (−8,266; 73,962)	1,419 (−14,037; 16,875)	144,116 (−38,465; 326,698)
Germany	25,716 (−9,387; 60,818)	−587 (−14,241; 13,068)	75,637 (37,244; 114,031)	17,789 (2,294; 33,284)	77,944 (35,559; 120,329)	24,046 (6,013; 42,078)	56,368 (8,607; 104,128)	8,806 (−12,422; 30,033)	285,718 (53,667; 517,770)
Luxembourg	−198 (−647; 250)	37 (−291; 366)	−294 (−1,006; 417)	285 (−231; 801)	−238 (−1,250; 775)	−8 (−729; 712)	−339 (−1,675; 998)	−37 (−987; 912)	−793 (−6,816; 5,230)
Netherlands	7,503 (2,353; 12,654)	1,736 (−3,037; 6,509)	12,707 (6,655; 18,758)	8,674 (3,148; 14,200)	11,819 (4,608; 19,031)	8,109 (1,585; 14,632)	10,550 (2,050; 19,050)	4,485 (−3,227; 12,197)	65,584 (14,136; 117,032)
**Total**	**291,806 (59,723; 523,889)**	**86,548 (−443; 173,539)**	**659,282 (390,134; 928,430)**	**240,961 (132,844; 349,078)**	**378,032 (66,622; 689,443)**	**124,135 (−8,720; 256,990)**	**180,808 (−179,940; 541,556)**	**35,523 (−125,277; 196,323)**	**1,997,095 (334,943; 3,659,247)**

Each cell contains a point estimate and a 95% confidence interval in brackets. CEE, Central and Eastern Europe; NE, Northern Europe; SE, Southern Europe; WE, Western Europe. Bold font represents total values for countries within each of four European regions.

The age-standardised excess years of potential productive life lost per 100,000 population (eYPPLL_R) for all years and countries combined was 181 (CI: 166–197) and was more than double in men (252; CI: 231–273) than in women (102; CI: 93–112; Supplementary Table S3). The measure of productive life lost was the highest in 2021 (324; CI: 294–354, both sexes combined), reflecting this year’s greatest mortality surge. Similarly to excess deaths and eYLL_R measures, eYPPLL_R was the lowest in 2023 (82; CI: 39–126), less than half its highest value in 2021.

**Figure 3:**
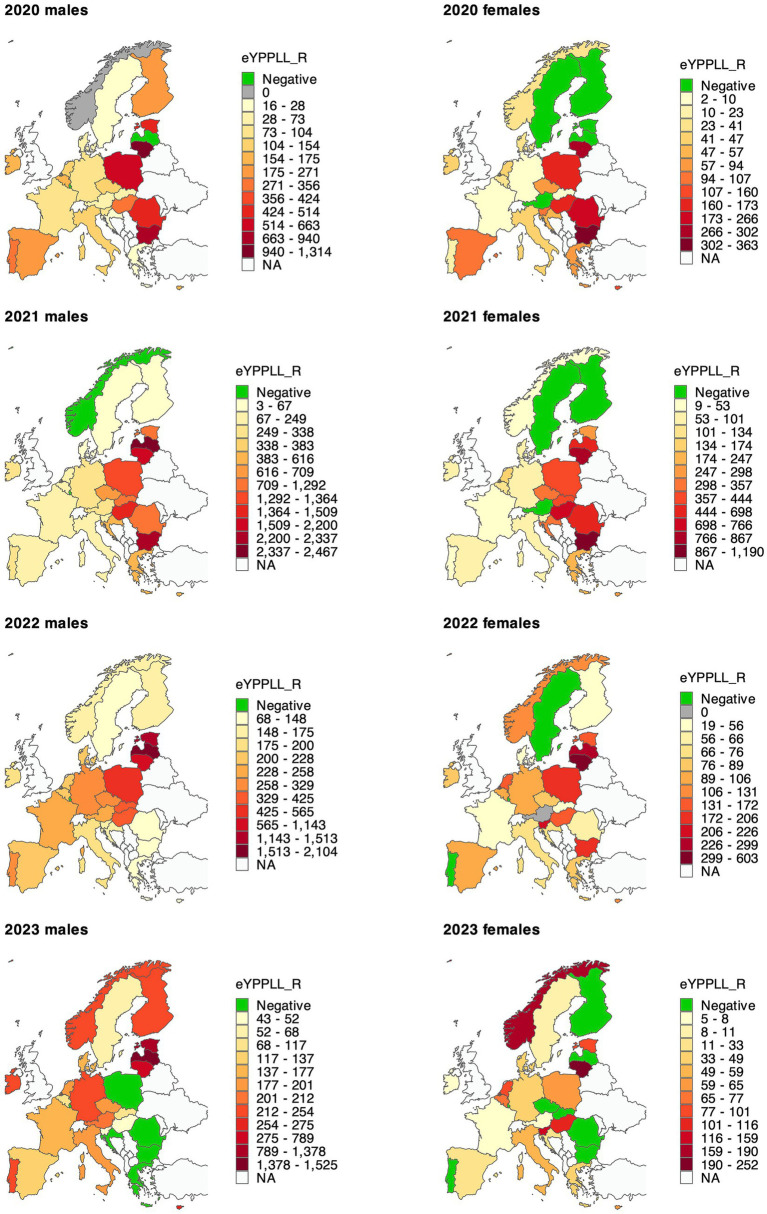
Age-standardised excess years of potential productive life lost per 100,000 working population in 28 countries in 2020–2023. Detailed results with confidence intervals are available in Supplementary Table S3. eYPPLL_R—age-standardised excess years of potential productive life lost rate per 100,000 population. Each map uses its own colour scale because the magnitude of excess years of potential productive life lost varies substantially across years (e.g., 2021 shows much higher values than 2023) and sexes.

For regional comparison of productive life lost, we note that CEE countries had the highest values of eYPPLL_R in 2020–2022 (Figure 3; Supplementary Table S3), and this was similar to the other two measures discussed above. However, in 2023, this region was the least burdened, with reduced, but non-significant, rates of excess productive years lost. For particular countries, the highest four-year eYPPLL_R was observed in Lithuania (956; CI: 566–1,347), Bulgaria (783; CI: 704–863), and Latvia (610; CI: 382–839), and the lowest in Luxembourg (−34; CI: −176–108), Sweden (7; CI: −62–77), and Norway (54; CI: −48–155).

For sex-specific eYPPLL_Rs, the four-year rate of excess productive years lost was the highest in Lithuania, both among men (1,427; CI: 794–2,059) and women (513; CI: 356–669). The burden of productive life lost in excess was skewed towards CEE and three Baltic states (Estonia, Latvia, and Lithuania), even more than in the other two measures.

### 3.5 Age-specific excess mortality burden

An age gradient was evident across all three excess mortality measures, with excess mortality identified primarily in age groups >60 years (the results in this sub-section refer to all countries combined). This trend was universal in eD for all 5-year age groups above 60 and both sexes in 2020–2022 (Supplementary Table S4). In 2023, the trend continued with some exceptions; for both sexes aged 60–64, we identified negative eD. Additionally, a reduced mortality was identified in the youngest age groups (0–4, 5–9, and 10–14), mainly in the first pandemic year. In all age groups but the eldest (85+), men account for a majority of eD. In all countries combined, 31.8% of eD were among those aged 85+, with a notable variation between sexes (24.8% in males and 40.4% in females). On the other hand, 12.2% of eD were identified in those below 65 years; this share was 16.4% for men and 7.1% for women (Supplementary Table S5).

For eYLL, the age groups with the most years lost were 65–69 and 75–79, not 85 + as in excess deaths. Again, years lost due to male mortality dominated, apart from those aged >80, where eYLL was higher in women (Supplementary Table S6). Across all countries, 42.3% of total eYLL were among those aged 75 + (34.8% in males and 52.8% in females; Supplementary Table S7). For the non-old population (aged <60), the share of total eYLL was 23.8% and was higher in males (29.6%) than in females (15.9%). Generally, the share of eYLL from the deaths of those aged <60 increased with time in both men (2020: 20.0%; 2023: 44.7%) and women (2020: 9.8%; 2023: 14.1%).

As for working-age population losses, the most burdened age groups were 40–44, 45–49, and 50–54 (Supplementary Table S8), with those aged <45 and 45 + contributing around 42 and 58% of all eYPPLL across all the countries and years, respectively. Similarly to years lost in the general population, we note a time-increasing share of losses in the younger sub-population (aged <45); in 2020, this group contributed 31.2% of total eYPPLL in males and 31.8% in females, while in 2023, the respective shares were 64.4% in males and 80.9% in females (Supplementary Table S9).

### 3.6 Comparison of excess and COVID-19 deaths

**Figure 4:**
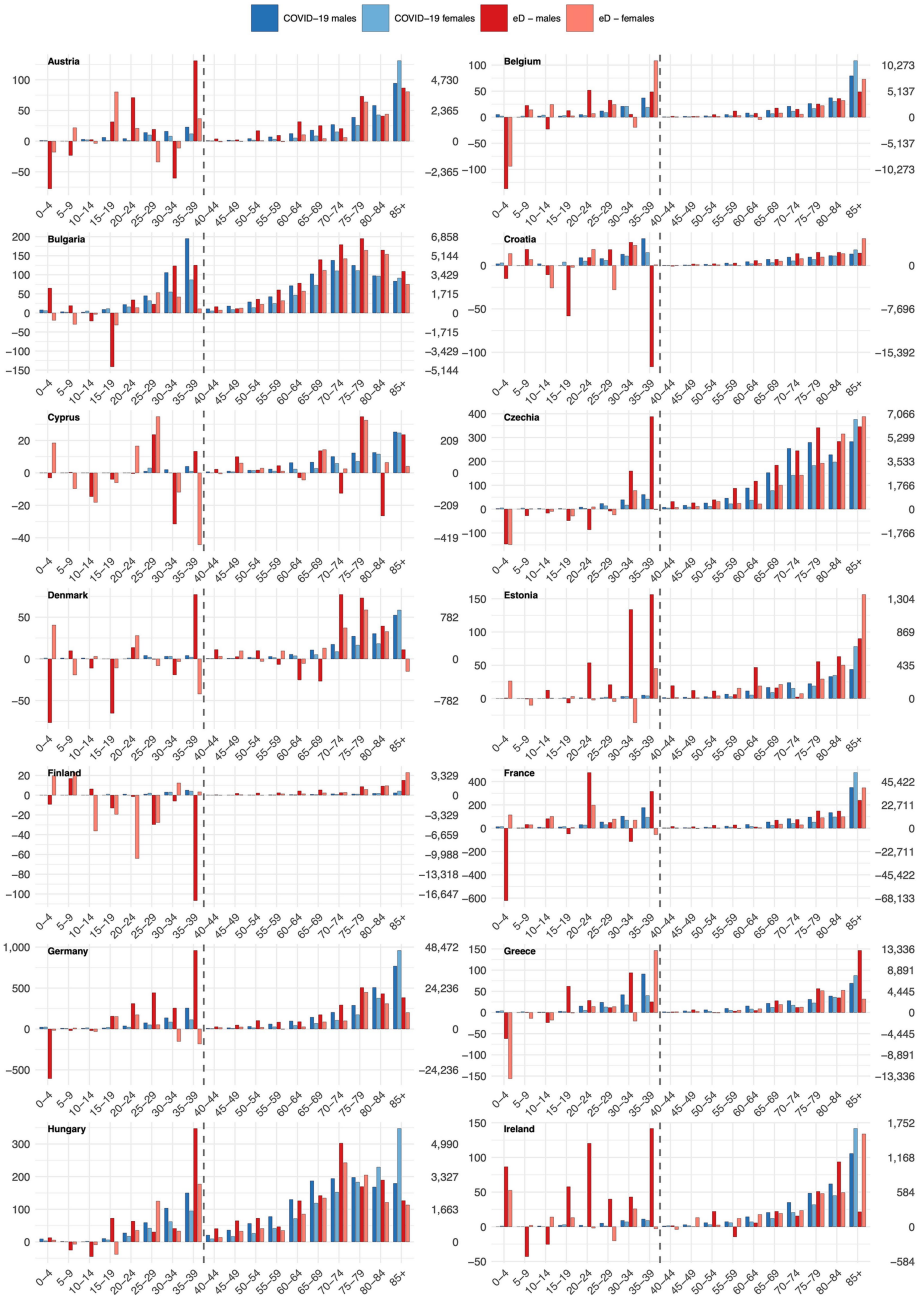
Comparison of excess deaths (eD) and COVID-19 deaths across 5-year age groups, part 1/2. The figure uses two separate scales; the left one for the younger (0–39 years) and the right one for the older (40–85+). This figure illustrates age-specific differences in cumulative excess mortality and COVID-19 mortality over the entire 2020–2023 period. Annual variations and early/late pandemic differences are only exhibited for excess mortality in Figures 1–3 and Tables 1–3.

**Figure 5:**
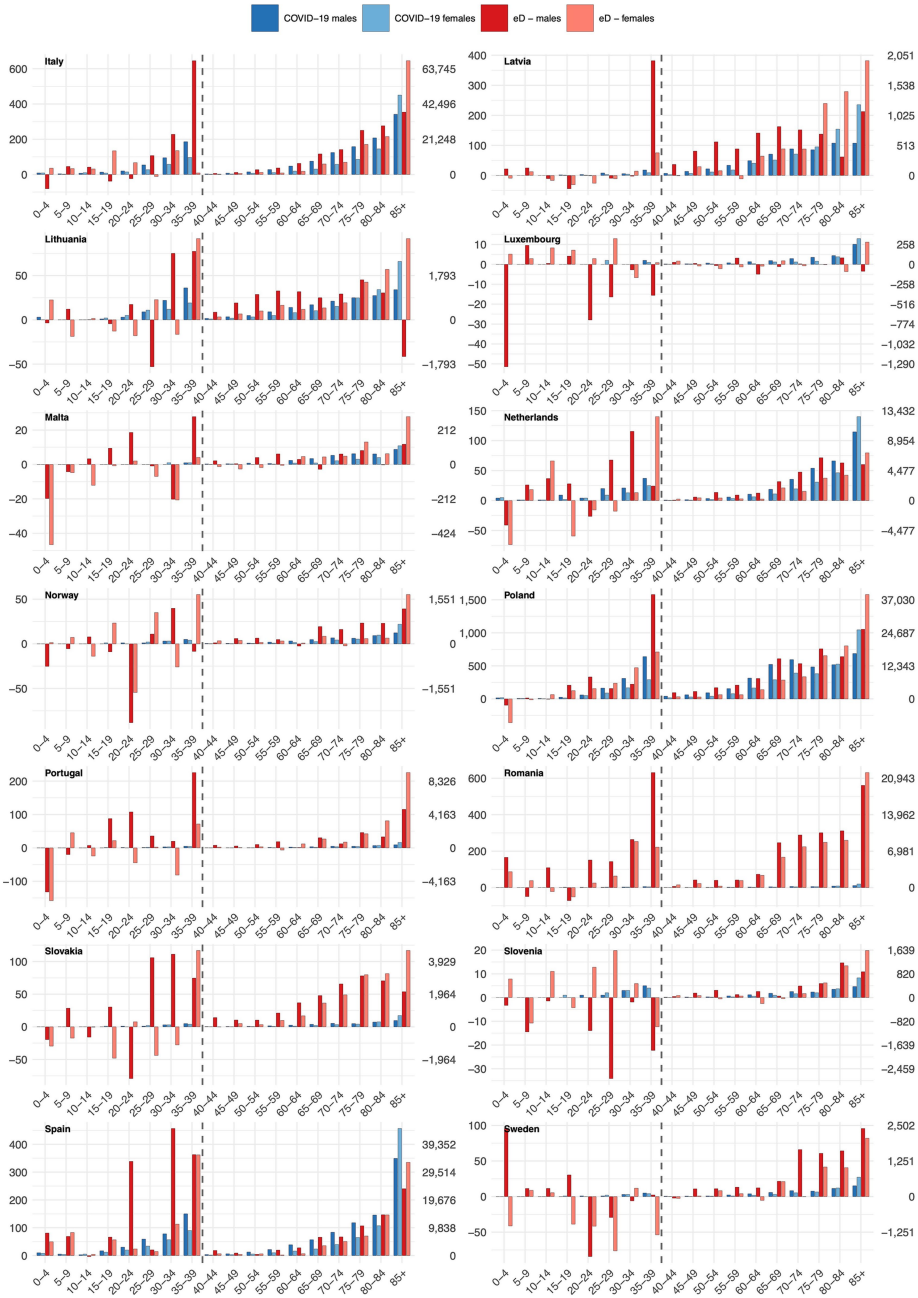
Comparison of excess deaths (eD) and COVID-19 deaths across 5-year age groups, part 2/2. The figure uses two separate scales; the left one for the younger (0–39 years) and the right one for the older (40–85+). This figure illustrates age-specific differences in cumulative excess mortality and COVID-19 mortality over the entire 2020–2023 period. Annual variations and early/late pandemic differences are only exhibited for excess mortality in Figures 1–3 and Tables 1–3.

Figures 4, 5 compare the number of excess deaths with the number of deaths due to COVID-19 in five-year age groups. We note notable differences among countries in comparing the two mortality measures. For countries as Belgium, Czechia, France, Ireland, or Spain, the COVID-19 deaths constitute a notable proportion of excess deaths (particularly in the older population), while in other territories as Finland, Portugal, Romania or Slovakia, the share of fatalities with a COVID-19 diagnosis assigned was very low. Additionally, the comparison shows that in some countries and age groups, particularly among the youngest (0–4, 5–9, 10–14, 15–19), the COVID-19 pandemic had a protective effect, reflected in reduced mortality. For example, in Sweden, we observed reduced deaths in the age groups 20–24, 25–29, 30–34 and 35–39.

### 3.7 Results of sensitivity analysis

To assess the robustness of expected mortality rate estimates, we compared the final specification (S2: short-baseline linear trend) with three alternative OLS (S1, S3, S4) and two time-series specifications (ARIMA and ETS), summarising absolute percentage deviations in age groups (Supplementary Table S10). Deviations for S1, S3, ARIMA and ETS were generally modest, with median differences around 3–5%. In contrast, the short-baseline quadratic model (S4) showed systematically larger discrepancies and substantially wider IQRs. As expected, deviations were greatest in age groups with very low mortality, where small absolute differences translate into large proportional changes.

A consistent age gradient emerged; in the 0–24 group, median deviations ranged from 8 to 13% for the linear and time-series models but exceeded 23% for S4. In working ages (25–64), deviations were considerably smaller, with S4 again showing the largest divergence (median 7.2%). At older ages (65+), all models remained close to the baseline, indicating stable forecasts where death counts are higher. Overall, these patterns show that quadratic specifications, especially S4, are prone to instability in low-mortality groups, while linear and time-series models remain close to the chosen baseline.

For life-years metrics, projected 2050 LE increased total eYLL by 15.5%, with proportionally larger rises in CEE and smaller changes in SE. These shifts affected magnitudes but did not alter the regional ranking of excess life-years lost. For eYPPLL, restricting productive ages to 20–64 changed results only marginally (+2.2% overall), with most countries remaining within ±10% of the baseline (Supplementary Table S11). Both sets of metrics, therefore, remain qualitatively stable across alternative assumptions.

## 4 Discussion

Our analysis of 28 European countries revealed 1.54 million excess deaths (eD), 16.7 million excess years of life lost (eYLL), and 2.0 million excess years of potential productive life lost (eYPPLL) between 2020 and 2023. The highest standardised excess mortality rates were observed in Central and Eastern Europe, particularly in Bulgaria, Romania, Slovakia, Poland, Lithuania and Latvia, while the lowest were in the Nordic countries (Norway and Denmark). In general, men experienced a higher excess mortality burden than women, and this difference was more pronounced in eYLL and eYPPLL, highlighting a higher mortality burden in younger-aged men. These findings are consistent with those reported elsewhere, regardless of the estimation method and data sources used (15, 17, 41–42).

The analysis by Pizzato et al. (17) showed a similar number of eD between 2020 and 2023. In absolute terms, the countries most burdened by excess mortality in our study align with those identified in (17) (Italy, Poland, Germany, France and Spain in the same order in both studies). Our estimate of eYLL is akin to the 16.8 million person-years of life lost (PYLL) reported for 18 European countries (41); however, although the results are very similar, they are not directly comparable because of differences in geographical scope (28 vs. 18 countries), methodology (all ages vs. population aged 35 and over, and forecasting approach), and the inclusion of the full year 2023. To benchmark our estimates further, Supplementary Table S12 compares our excess deaths with several influential multi-country studies, covering three reference periods: 2020 only, 2020–2021, and 2020–2023. For the most comparable interval, 2020–2021, our findings are broadly consistent with those reported by others, with notable differences concentrated in a few countries such as Denmark, Norway, and Sweden, where small absolute changes in mortality and differing baseline specifications can lead to appreciable variation across studies. Our estimate for Germany in 2020–2021 (64,619 eD) is also lower than several published estimates, which range 88–203 thousand eD (Supplementary Table S12). However, our figure aligns with the age-adjusted estimate of Levitt et al. (14), who reported 54,740 eD for the same period. In contrast, their unadjusted estimate was 128,557 deaths, underscoring that age composition plays a substantial role in excess mortality calculations. The close correspondence between our 5-year age-group modelling framework and the age-adjusted results of Levitt et al. supports the validity of our age-structured approach. Therefore, the greatest divergence across studies was identified for countries with small populations or low excess mortality (e.g., Denmark, Norway, Sweden, Luxembourg, Malta), where even minor differences in baseline specification or age adjustment can generate large discrepancies. In contrast, estimates were much more consistent for large countries such as Italy, France, Poland, and Spain, reinforcing the stability of excess mortality calculations at larger scales.

Among Europeans of working age (20–64 years), the total eYPPLL was 1,997,095, representing 12.0% of the total eYLL, highlighting the significant impact of the pandemic on mortality among those aged 65 and older. We found no studies evaluating the years of potential productive life lost due to excess mortality in the working population caused by COVID-19. This emphasises the importance of our study in understanding COVID-19’s effects on working-age individuals.

The higher excess mortality among men aligns with the findings of Beegle et al. (44), which showed that in high-income countries, men died on average 2.2 times more often in 2020 and 1.8 times in 2021; yet, the magnitude of the variation depended on age structure and comorbidity burden. Other studies also support this unfavourable tendency in men (12, 41–43, 45, 46). An additional interesting finding from (44) is that the average male-to-female mortality ratio was higher for eD than for expected all-cause deaths in both 2020 and 2021. Our data suggest that this pattern extends beyond the number of deaths; specifically, the number of eD in men is 21.4% higher than in women, the number of eYLL in men is 38.1% higher, and the number of eYPPLL in men is 209.9% higher. This means that more men died at younger ages, and the difference was particularly strong at working ages. Possible explanations include biological differences (such as immune response), a higher prevalence of risk factors (smoking, alcohol intake and chronic diseases), and greater occupational exposure (47–52). However, as pointed out previously (46), differences in excess mortality between males and females are not unique to the COVID-19 pandemic but are characteristic of any excess mortality.

The greatest losses were identified in the age groups 80–84 and 85 + for eD, 65–69 and 75–79 for eYLL, and 45–49 and 50–54 for eYPPLL. Compared to (41), our study shows notable differences in how years of life lost are distributed; in our analysis, 42.3% of total eYLL was in the 75 + age group, while the younger population (under 60) accounted for 23.8%, which sharply contrasts with the 30% of total years of life lost in the 65–80 age group and 60% in those aged 80 and older in (41). Our detailed age-specific findings show that COVID-19 does not spare the working-age population. Although most deaths occurred among the older population, a significant burden was identified in younger age groups, especially those aged 40–54. Also, the burden in the younger population was geographically disproportionate, with CEE and Baltic states (Latvia, Lithuania and Estonia) experiencing the heaviest death toll. This crucial finding calls for further analysis of excess mortality among the working population, since European states face severe labour shortages undermining economic growth (16).

After a record high mortality in 2021, the excess rates declined but remained above zero in most countries in 2023. Although the number of eD in 2023 was 77% lower than in 2020, this situation highlights that the ongoing indirect pandemic’s impacts persist, even with widespread COVID-19 vaccination efforts (53). The results of the cross-country study (17) also support our findings, showing the highest burden in 2021. The other research (41) indicated that the years of life lost due to COVID-19 decreased over time since the start of the pandemic, while the years of life lost from other causes increased. A regional study (42) showed that in 2021, the highest cumulative loss of life years was almost entirely concentrated in CEE, while some Western regions experienced gains in life years. A study covering 13 WE countries (54) identified significant variations in cumulative mortality between the countries analysed—those with faster non-pharmaceutical interventions (NPIs) responses and higher vaccination enrollment (e.g., Denmark) had lower age- and sex-standardised cumulative excess mortality. The link between timely vaccination coverage and reduced excess mortality is well documented. Another cross-European study (53) found that the average excess mortality in the countries with slower vaccination rollout (less than 70% vaccination coverage by January 2022) was nearly five times higher than in those with faster rollout (more than 70% vaccination coverage by January 2022).

Our results confirm geographic differences in the pandemic burden. The largest East–West gradient was observed in 2021, consistent with the results of the studies by Bonnet et al. (42) and Pizzato (17). In 2022, the gradient between East and West countries persisted, but in 2023, the situation changed. We identified the negative excess mortality in 2023 in countries that were among the most burdened in 2020–2022 (Bulgaria, Hungary, Romania, and Lithuania, among others). This gradient might result from worse structural and psychosocial factors in the CEE post-transitional countries, leading to higher mortality therein (55). A magnitude of socio-economic factors was outlined in the study (17) which found that lower GDP per capita, higher poverty levels, and lower healthcare spending correlated with greater excess mortality, while vaccination coverage had a protective effect on mortality.

The East–West mortality divergence, the persistence of excess mortality in most countries in 2023, and the negative excess mortality in 2023 in countries heavily affected during the COVID-19 pandemic may stem from several factors. Firstly, a sustained increase in mortality following the pandemic was related to long-term health complications caused by the virus (56, 57), but also disruptions in healthcare services such as missed cancer screenings and delayed surgeries (58–60). Additionally, emerging mental health issues resulting from social isolation measures and pandemic-borne fears plausibly played a role (61). Secondly, the harvesting effect suggests that the virus mainly impacted the most vulnerable individuals, potentially resulting in decreased mortality in the post-pandemic period (7). Thirdly, the reverse harvesting effect (7) or dry tinder hypothesis (62) suggest that at the pandemic onset, NPIs protected the most vulnerable from seasonal infections that could have led to death. In later years, as restrictions were eased, an increased mortality was observed among the most vulnerable due to infections other than COVID-19.

As for the comparison of excess deaths and COVID-19 deaths in particular countries, the differences identified in our study are in line with Sanmarchi et al.’s study (63) showing that countries differ notably in their capabilities to test, diagnose, and certify COVID-19 deaths. Therefore, excess mortality exceeded confirmed COVID-19 mortality, particularly in countries with lower testing capacity or more restrictive diagnostic practises, including several Eastern European countries or Portugal, among others. On the other hand, countries with more efficient certification systems and broader inclusion of suspected COVID-19 deaths, such as Belgium, France or Spain, showed smaller gaps between the two mortality measures.

The sensitivity analyses indicate that our findings are robust to alternative modelling and conceptual assumptions. Alternative linear and time-series models produced only minor deviations from the selected specification, but quadratic trends, particularly using short baseline, showed instability in low-mortality young age groups, reinforcing the choice of a linear specification. Likewise, although extending LE assumptions increased absolute eYLL levels, and adjusting productive-age ranges slightly shifted eYPPLL, these variations did not alter the relative ranking of countries. Together, these results demonstrate that our findings are not sensitive to modelling choices or scenario assumptions.

Before we conclude, we shall acknowledge the strengths and limitations of our analysis. For strengths, we analysed a comprehensive set of excess mortality measures, including excess death counts, eYLL and eYPPLL, capturing not only the epidemiological burden of excess deaths but also the economic dimension of these losses. Also, we used detailed 5-year age groups, unravelling patterns unobservable in studies using all-age or wide age intervals. Additionally, we analysed a set of 28 countries through the year 2023, allowing for assessment of the post-pandemic situation and relied on complete and final death statistics from Eurostat, not short-term data that requires verification. Moreover, we reduced arbitrariness in baseline selection by evaluating multiple baseline windows and trend specifications and selecting the final forecasting model using selection criteria. Eventually, we evaluated model robustness using alternative specifications and forecasting methods through sensitivity analysis, supporting the stability of our findings.

Several limitations should be acknowledged. First, we used annual data, which limited modelling choices, leading to reliance on annual OLS trend models evaluated across multiple baseline windows and specifications. Consequently, the confidence intervals for our estimates are fairly wide; yet, this shortcoming is not unique to our study, as several prior analyses reported results of even larger uncertainty (15, 25, 32). Second, the estimates for low-populated countries should be treated with caution, as the small number of deaths leads to greater statistical uncertainty and increased risk of random variation affecting the estimates. Third, we used all-cause mortality data and did not account for the causes of death. Fourth, the methodological choice regarding eYLL and eYPPLL might have affected our findings; therefore we addressed this last limitation through sensitivity analysis. Fifth, aggregated estimates implicitly treat forecast errors across age groups as independent, which may lead to conservative uncertainty intervals. Sixth, although we accounted for mortality-model uncertainty when estimating eYLL and eYPPLL, additional uncertainty arising from future life expectancy changes or evolving labour market participation patterns was not formally quantified and was therefore addressed only through scenario-based analyses. Finally, excess mortality in 2023 may partly reflect non-COVID factors, such as heatwaves, influenza resurgence, or health systems disruptions, which were not examined in this study and may contribute to the observed post-pandemic mortality burden.

## 5 Conclusion

Our results show that COVID-19 has exacerbated existing demographic inequalities in Europe, particularly between East and West and between sexes. Although the pandemic mortality burden has generally decreased, it was still identified in 2023, requiring further preventive and systemic actions. The persistence of excess mortality in 2023 likely reflects a combination of long-term pandemic effects and non-COVID factors. Integrating health and economic indicators, such as the eD, eYLL and eYPPLL, should become an element of public health policy to better recognise different dimensions of mortality burden and to prepare for future health crises.

As for health policy implications, CEE should prioritise efforts towards improving their health care systems and vaccination acceptance to limit excess health burden in future. Further, the countries should maintain monitoring efforts; although excess rates are declining, they remained positive in 2023 in several states, underscoring the need for long-term surveillance of the pandemic’s consequences. Importantly, our estimates of productive-life losses show that the economic burden of the pandemic is substantial. The future research efforts should assess this burden in monetary terms to inform policymakers on how much Europe lost through production uncompleted due to excess mortality, as cross-country evidence in this respect is scarce (64).

## Data Availability

Publicly available datasets only were analysed in this study. This data can be found here: Eurostat databases (18–20).
