# Supplementary material for: Beyond death counts: how COVID-19 affected excess mortality and productive life-years lost across 28 European states, 2020–2023
Source: Front Public Health. 2026 Jan 20;13:1720864. doi: 10.3389/fpubh.2025.1720864 (PMC12864430; doi:10.3389/fpubh.2025.1720864)
Supplement: Supplementary file 1 [file Data_Sheet_1.PDF]

Supplementary file

Beyond death counts: how COVID-19 affected excess mortality and productive life-years lost across 28 European states, 2020–2023

Paweł Niewiadomski[1], Jakub Wojtasik[2], Błażej Łyszczarz[3]

1. Doctoral School of Medical and Health Sciences, Nicolaus Copernicus University in Toruń, Bydgoszcz, Poland. e-mail: pawel.niewiadomski@doktorant.umk.pl

2 Statistical Analysis Centre & Doctoral School of Social Sciences, Nicolaus Copernicus University in Toruń, Poland. e-mail: jwojtasik@umk.pl

3. Department of Health Economics, Nicolaus Copernicus University in Toruń, Bydgoszcz, Poland. e-mail: blazej@cm.umk.pl

TABLE S1. AGE-STANDARDISED EXCESS MORTALITY RATE PER 100,000 POPULATION IN 28 COUNTRIES IN 2020-2023 2

TABLE S2. AGE-STANDARDISED EXCESS YEARS OF LIFE LOST PER 100,000 POPULATION IN 28 COUNTRIES IN 2020-2023 4

TABLE S3. AGE-STANDARDISED EXCESS YEARS OF POTENTIAL PRODUCTIVE LIFE LOST PER 100,000 WORKING POPULATION IN 28 COUNTRIES IN 2020-2023 6

TABLE S4. EXCESS DEATHS IN 5-YEAR AGE GROUPS IN 28 EUROPEAN COUNTRIES IN 2020-2023 8

TABLE S5. SHARE OF EXCESS DEATHS IN SELECTED AGE-SPECIFIC SUB-POPULATIONS IN 28 EUROPEAN COUNTRIES IN 2020-2023 9

TABLE S6. EXCESS YEARS OF LIFE LOST (EYLL) IN 5-YEAR AGE GROUPS IN 28 EUROPEAN COUNTRIES IN 2020-2023 10

TABLE S7. SHARE OF EXCESS YEARS OF LIFE LOST (EYLL) IN SELECTED AGE-SPECIFIC SUB-POPULATIONS IN 28 EUROPEAN COUNTRIES IN 2020-2023 11

TABLE S8. EXCESS YEARS OF POTENTIAL PRODUCTIVE LIFE LOST (EYPPLL) IN 5-YEAR AGE GROUPS IN 28 EUROPEAN COUNTRIES IN 2020-2023 11

TABLE S9. SHARE OF EXCESS YEARS OF POTENTIAL LIFE LOST (EYPPLL) IN SELECTED AGE-SPECIFIC SUB-POPULATIONS IN 28 EUROPEAN COUNTRIES IN 2020-2023 11

TABLE S10. SENSITIVITY ANALYSIS: ABSOLUTE PERCENTAGE DEVIATIONS FROM BASELINE MODEL (S2), BY AGE GROUP AND MODEL SPECIFICATION 12

TABLE S11. SENSITIVITY ANALYSIS: EXCESS YEARS OF LIFE LOST (EYLL) AND EXCESS YEARS OF POTENTIAL PRODUCTIVE LIFE LOST (EYLL) UNDER ALTERNATIVE ASSUMPTIONS ON LIFE EXPECTANCY AND PRODUCTIVE AGES 13

TABLE S12. CROSS-COUNTRY COMPARISON OF EXCESS DEATHS ESTIMATES FROM MAJOR INTERNATIONAL STUDIES AND THIS ANALYSIS 14

MISSING DATA IMPUTATION 16

TABLE S13. DETAILS OF DATA COVERAGE 18

Table S1. Age-standardised excess mortality rate per 100,000 population in 28 countries in 2020-2023

	2020			2021			2022			2023			2020-2023		
Country	Total	Males	Females	Total	Males	Females	Total	Males	Females	Total	Males	Females	Total	Males	Females
**CEE**	**171 (157; 184)**	**267 (251; 282)**	**116 (105; 127)**	**378 (362; 394)**	**542 (523; 561)**	**275 (263; 288)**	**118 (99; 137)**	**175 (152; 198)**	**86 (71; 101)**	**-23 (-45; 0)**	**-24 (-51; 3)**	**-18 (-36; -1)**	**190 (182; 198)**	**285 (276; 295)**	**135 (129; 142)**
Bulgaria	222 (211; 234)	334 (326; 343)	150 (137; 163)	571 (558; 584)	754 (742; 765)	452 (438; 467)	135 (120; 150)	208 (194; 222)	94 (78; 110)	-105 (-122; -87)	-121 (-138; -104)	-92 (-110; -75)	257 (239; 274)	374 (357; 390)	184 (167; 202)
Czechia	164 (151; 177)	232 (215; 248)	120 (108; 131)	271 (256; 286)	393 (375; 412)	188 (175; 200)	88 (71; 106)	125 (104; 146)	66 (51; 81)	0 (-20; 20)	23 (0; 47)	-12 (-30; 5)	153 (132; 174)	218 (191; 245)	110 (93; 126)
Hungary	107 (97; 117)	143 (131; 155)	86 (77; 94)	254 (241; 266)	348 (333; 363)	193 (182; 203)	62 (46; 77)	95 (76; 114)	43 (29; 56)	-18 (-36; 0)	4 (-20; 28)	-23 (-38; -7)	122 (103; 142)	174 (149; 199)	91 (77; 106)
Poland	182 (172; 193)	277 (263; 291)	124 (116; 133)	325 (312; 337)	447 (430; 464)	246 (236; 256)	126 (110; 141)	170 (151; 190)	98 (85; 111)	6 (-12; 24)	12 (-11; 36)	6 (-10; 21)	187 (168; 205)	264 (239; 289)	138 (125; 152)
Romania	188 (172; 204)	283 (262; 303)	126 (113; 140)	405 (388; 422)	516 (494; 537)	331 (317; 346)	150 (131; 168)	228 (204; 252)	109 (92; 125)	-17 (-39; 5)	-4 (-31; 23)	-15 (-35; 4)	209 (188; 230)	293 (266; 319)	158 (141; 175)
Slovakia	117 (99; 135)	169 (146; 193)	88 (72; 103)	421 (401; 441)	545 (520; 571)	336 (318; 353)	166 (142; 189)	209 (179; 239)	135 (115; 155)	34 (6; 62)	41 (5; 76)	31 (7; 55)	201 (173; 229)	265 (227; 302)	159 (138; 180)
**NE**	**31 (19; 44)**	**45 (28; 62)**	**19 (11; 27)**	**72 (57; 86)**	**92 (73; 112)**	**53 (42; 63)**	**85 (67; 102)**	**101 (78; 123)**	**71 (59; 84)**	**43 (23; 64)**	**58 (32; 85)**	**31 (16; 47)**	**54 (47; 62)**	**71 (61; 82)**	**39 (34; 44)**
Denmark	-7 (-21; 6)	-1 (-16; 15)	-10 (-22; 3)	22 (5; 39)	26 (5; 47)	21 (6; 35)	46 (25; 67)	49 (22; 76)	44 (27; 62)	12 (-14; 38)	15 (-19; 48)	9 (-12; 30)	13 (-10; 37)	17 (-11; 45)	12 (-8; 32)
Estonia	20 (4; 36)	30 (6; 53)	13 (1; 26)	218 (199; 237)	326 (298; 355)	160 (146; 174)	135 (112; 157)	216 (182; 250)	89 (71; 106)	41 (14; 67)	104 (63; 145)	12 (-9; 32)	99 (68; 129)	157 (113; 202)	66 (44; 88)
Finland	12 (3; 22)	17 (3; 32)	6 (-1; 12)	39 (28; 50)	55 (39; 70)	27 (19; 35)	116 (103; 129)	143 (125; 161)	96 (87; 106)	87 (72; 102)	118 (98; 138)	65 (54; 77)	52 (35; 69)	71 (47; 96)	36 (25; 47)
Ireland	11 (-6; 28)	5 (-17; 27)	15 (1; 28)	51 (32; 70)	57 (33; 82)	43 (28; 58)	60 (39; 81)	46 (19; 73)	68 (51; 84)	52 (29; 75)	51 (22; 80)	52 (33; 71)	39 (17; 61)	36 (8; 64)	39 (22; 57)
Latvia	49 (35; 63)	78 (59; 98)	35 (23; 47)	341 (324; 357)	470 (448; 492)	259 (245; 274)	178 (159; 198)	270 (245; 296)	118 (100; 136)	53 (29; 76)	112 (83; 142)	11 (-11; 32)	155 (127; 182)	233 (197; 270)	107 (85; 128)
Lithuania	174 (155; 194)	271 (237; 305)	114 (102; 126)	294 (269; 319)	311 (264; 357)	256 (240; 272)	154 (121; 187)	114 (58; 169)	147 (124; 171)	-37 (-78; 3)	-124 (-189; -59)	-21 (-50; 9)	177 (136; 217)	195 (130; 260)	145 (121; 169)
Norway	-8 (-19; 3)	-2 (-20; 16)	-12 (-18; -6)	16 (3; 29)	16 (-3; 36)	15 (7; 22)	87 (72; 102)	117 (95; 139)	63 (54; 73)	42 (25; 59)	59 (35; 83)	28 (16; 39)	26 (7; 45)	40 (12; 67)	14 (2; 25)
Sweden	67 (58; 75)	95 (83; 107)	45 (39; 51)	11 (0; 22)	27 (12; 41)	-2 (-11; 7)	29 (14; 44)	39 (22; 57)	19 (6; 33)	18 (-1; 38)	28 (6; 49)	10 (-7; 28)	39 (24; 55)	56 (36; 75)	26 (15; 37)
**SE**	**98 (87; 108)**	**115 (102; 129)**	**82 (75; 90)**	**96 (84; 108)**	**127 (111; 143)**	**70 (61; 79)**	**85 (70; 99)**	**104 (86; 123)**	**71 (60; 83)**	**23 (6; 40)**	**32 (10; 53)**	**19 (5; 32)**	**84 (78; 91)**	**104 (96; 113)**	**69 (64; 74)**
Croatia	114 (101; 128)	151 (132; 170)	90 (79; 101)	266 (250; 281)	348 (327; 369)	209 (196; 222)	149 (130; 168)	184 (160; 208)	125 (109; 141)	20 (-2; 43)	39 (11; 67)	6 (-14; 25)	151 (129; 174)	197 (168; 226)	120 (102; 138)
Cyprus	3 (-14; 19)	-3 (-19; 14)	7 (-10; 24)	83 (58; 107)	103 (77; 129)	68 (44; 92)	84 (52; 116)	81 (46; 115)	88 (57; 119)	-16 (-56; 24)	-23 (-67; 20)	-8 (-47; 30)	32 (2; 62)	31 (-2; 63)	34 (7; 61)
Greece	43 (31; 56)	53 (38; 68)	36 (24; 47)	138 (122; 154)	177 (159; 194)	107 (93; 122)	115 (95; 134)	164 (143; 185)	84 (65; 102)	26 (3; 49)	65 (41; 89)	4 (-19; 27)	79 (61; 97)	108 (88; 129)	59 (43; 74)

Italy	123 (114; 132)	164 (151; 177)	91 (85; 96)	82 (72; 92)	107 (93; 122)	61 (54; 68)	94 (82; 106)	113 (96; 129)	79 (71; 88)	43 (29; 56)	51 (32; 70)	35 (25; 45)	93 (80; 106)	118 (99; 137)	73 (64; 81)
Malta	62 (44; 79)	80 (59; 101)	40 (25; 54)	59 (37; 81)	39 (11; 67)	67 (49; 85)	58 (31; 86)	68 (31; 104)	46 (24; 67)	29 (-3; 61)	24 (-20; 68)	31 (6; 56)	57 (26; 88)	62 (25; 100)	47 (20; 73)
Portugal	71 (60; 83)	81 (67; 96)	63 (53; 73)	74 (61; 87)	92 (75; 108)	60 (50; 71)	61 (46; 75)	58 (40; 77)	60 (48; 72)	13 (-4; 29)	7 (-14; 29)	15 (2; 29)	61 (43; 78)	68 (45; 91)	54 (40; 68)
Slovenia	142 (129; 154)	178 (160; 196)	118 (109; 128)	103 (88; 119)	154 (130; 177)	67 (55; 78)	61 (42; 80)	68 (38; 98)	53 (39; 67)	11 (-12; 34)	31 (-5; 68)	-4 (-20; 13)	101 (79; 123)	134 (102; 165)	79 (64; 94)
Spain	127 (119; 136)	163 (153; 173)	101 (94; 108)	50 (40; 60)	75 (63; 86)	32 (23; 41)	66 (53; 79)	81 (67; 95)	55 (43; 67)	15 (-1; 31)	15 (-2; 32)	15 (0; 30)	78 (66; 91)	100 (84; 116)	62 (52; 72)
**WE**	**66 (56; 75)**	**89 (76; 102)**	**47 (41; 54)**	**57 (46; 68)**	**87 (72; 102)**	**34 (27; 42)**	**68 (55; 81)**	**88 (71; 106)**	**52 (43; 62)**	**27 (12; 43)**	**40 (20; 60)**	**18 (7; 29)**	**58 (52; 64)**	**81 (73; 89)**	**41 (37; 45)**
Austria	74 (66; 83)	104 (93; 116)	53 (46; 59)	74 (63; 85)	107 (94; 120)	48 (38; 58)	83 (70; 96)	114 (99; 129)	60 (48; 72)	38 (23; 53)	66 (48; 84)	19 (5; 32)	71 (55; 86)	101 (82; 121)	49 (37; 60)
Belgium	138 (127; 149)	173 (157; 188)	111 (103; 119)	37 (25; 49)	71 (55; 88)	11 (2; 20)	66 (53; 79)	76 (58; 94)	57 (47; 67)	26 (11; 41)	31 (11; 52)	21 (10; 33)	75 (58; 92)	97 (73; 121)	58 (47; 70)
France	63 (56; 69)	92 (82; 102)	43 (39; 46)	57 (50; 64)	87 (76; 97)	36 (31; 40)	68 (60; 75)	90 (78; 101)	51 (46; 56)	20 (11; 29)	26 (14; 39)	14 (8; 20)	55 (45; 65)	78 (63; 93)	38 (33; 44)
Germany	22 (13; 31)	38 (27; 48)	10 (3; 18)	46 (36; 57)	72 (60; 85)	26 (16; 36)	76 (63; 89)	101 (86; 115)	57 (45; 69)	31 (15; 46)	46 (30; 63)	18 (4; 33)	40 (26; 54)	61 (45; 77)	24 (12; 36)
Luxembourg	46 (34; 58)	68 (51; 85)	29 (20; 39)	11 (-8; 29)	8 (-18; 34)	8 (-6; 22)	-14 (-39; 11)	-43 (-80; -7)	2 (-16; 21)	-33 (-64; -1)	-89 (-136; -42)	4 (-19; 28)	24 (-3; 50)	28 (-7; 64)	19 (-1; 38)
Netherlands	78 (69; 88)	115 (102; 128)	50 (43; 58)	84 (72; 95)	119 (105; 133)	56 (45; 67)	66 (50; 81)	77 (60; 94)	54 (38; 71)	48 (29; 67)	61 (42; 80)	36 (16; 57)	74 (58; 89)	100 (80; 119)	51 (40; 63)
**Total**	**86 (80; 91)**	**127 (120; 135)**	**59 (55; 63)**	**126 (119; 133)**	**190 (181; 198)**	**81 (77; 86)**	**85 (77; 93)**	**112 (102; 122)**	**66 (61; 72)**	**21 (12; 30)**	**30 (18; 41)**	**15 (8; 22)**	**87 (84; 91)**	**127 (123; 132)**	**61 (58; 63)**

Notes: Each cell contains a point estimate and a 95% confidence interval in parentheses. CEE – Central and Eastern Europe; NE – Northern Europe; SE – Southern Europe; WE – Western Europe.

Table S2. Age-standardised excess years of life lost per 100,000 population in 28 countries in 2020-2023

	2020			2021			2022			2023			2020-2023		
Country	Total	Males	Females	Total	Males	Females	Total	Males	Females	Total	Males	Females	Total	Males	Females
**CEE**	**1,787 (1,598; 1,975)**	**2,545 (2,321; 2,770)**	**1,256 (1,110; 1,403)**	**4,405 (4,176; 4,634)**	**5,638 (5,362; 5,913)**	**3,456 (3,279; 3,634)**	**1,064 (790; 1,337)**	**1,443 (1,111; 1,775)**	**781 (567; 994)**	**-321 (-644; 1)**	**-373 (-765; 20)**	**-255 (-507; -4)**	**2,073 (1,954; 2,192)**	**2,787 (2,643; 2,930)**	**1,563 (1,470; 1,656)**
Bulgaria	2,457 (2,345; 2,570)	3,290 (3,178; 3,402)	1,832 (1,717; 1,946)	6,443 (6,305; 6,581)	7,618 (7,478; 7,758)	5,569 (5,430; 5,708)	1,292 (1,125; 1,459)	1,626 (1,451; 1,801)	1,108 (944; 1,271)	-901 (-1,101; -702)	-1,019 (-1,234; -805)	-799 (-992; -606)	2,930 (2,672; 3,188)	3,720 (3,456; 3,983)	2,366 (2,125; 2,607)
Czechia	1,319 (1,170; 1,468)	1,818 (1,629; 2,008)	938 (820; 1,055)	3,035 (2,868; 3,203)	4,066 (3,854; 4,278)	2,196 (2,064; 2,328)	802 (611; 993)	1,064 (826; 1,302)	585 (432; 738)	-79 (-296; 137)	106 (-159; 372)	-234 (-411; -58)	1,483 (1,187; 1,779)	2,013 (1,649; 2,376)	1,059 (827; 1,290)
Hungary	1,140 (972; 1,307)	1,401 (1,175; 1,628)	955 (839; 1,071)	3,491 (3,302; 3,679)	4,313 (4,055; 4,570)	2,861 (2,732; 2,989)	848 (633; 1,064)	1,095 (800; 1,391)	673 (525; 821)	-75 (-321; 172)	-73 (-416; 269)	-10 (-178; 159)	1,559 (1,224; 1,893)	1,940 (1,506; 2,374)	1,289 (1,048; 1,530)
Poland	1,930 (1,802; 2,057)	2,766 (2,596; 2,936)	1,302 (1,208; 1,396)	3,732 (3,584; 3,881)	4,820 (4,625; 5,015)	2,879 (2,768; 2,991)	1,280 (1,106; 1,453)	1,681 (1,459; 1,903)	958 (824; 1,092)	-79 (-279; 121)	-121 (-376; 134)	-23 (-179; 133)	2,009 (1,739; 2,279)	2,678 (2,324; 3,032)	1,492 (1,299; 1,686)
Romania	1,976 (1,846; 2,106)	2,684 (2,524; 2,845)	1,471 (1,359; 1,583)	4,433 (4,284; 4,582)	5,191 (5,005; 5,376)	3,877 (3,750; 4,004)	799 (625; 973)	1,248 (1,030; 1,466)	516 (371; 662)	-709 (-911; -508)	-842 (-1,096; -588)	-545 (-711; -379)	2,034 (1,759; 2,308)	2,600 (2,259; 2,940)	1,650 (1,438; 1,863)
Slovakia	1,075 (904; 1,247)	1,462 (1,230; 1,694)	819 (688; 951)	4,848 (4,656; 5,041)	6,058 (5,801; 6,315)	3,863 (3,714; 4,012)	1,391 (1,169; 1,613)	1,934 (1,640; 2,228)	928 (754; 1,102)	146 (-110; 403)	257 (-80; 594)	43 (-162; 247)	2,055 (1,695; 2,414)	2,657 (2,191; 3,123)	1,582 (1,314; 1,850)
**NE**	**313 (119; 506)**	**454 (207; 700)**	**158 (19; 297)**	**782 (549; 1,015)**	**1,017 (729; 1,305)**	**566 (389; 742)**	**851 (569; 1,133)**	**1,031 (692; 1,369)**	**688 (466; 911)**	**535 (199; 870)**	**711 (315; 1,106)**	**364 (91; 637)**	**573 (451; 696)**	**762 (611; 914)**	**384 (292; 477)**
Denmark	-44 (-214; 126)	-58 (-247; 131)	-15 (-169; 139)	211 (17; 405)	114 (-104; 331)	319 (147; 492)	512 (284; 741)	456 (199; 712)	572 (369; 775)	232 (-34; 499)	259 (-41; 559)	210 (-28; 448)	168 (-192; 527)	127 (-273; 526)	218 (-103; 540)
Estonia	337 (111; 563)	505 (193; 816)	164 (-1; 330)	2,613 (2,338; 2,888)	3,639 (3,281; 3,997)	1,830 (1,616; 2,045)	1,715 (1,379; 2,051)	2,651 (2,231; 3,071)	940 (662; 1,219)	883 (481; 1,284)	1,540 (1,050; 2,029)	344 (-2; 689)	1,281 (721; 1,841)	1,947 (1,231; 2,663)	744 (322; 1,166)
Finland	232 (95; 370)	471 (270; 673)	-18 (-99; 63)	389 (229; 548)	571 (345; 797)	219 (119; 318)	1,127 (940; 1,314)	1,353 (1,095; 1,610)	922 (798; 1,047)	905 (685; 1,125)	1,194 (899; 1,489)	643 (490; 795)	536 (232; 840)	784 (369; 1,199)	280 (82; 479)
Ireland	248 (107; 388)	213 (41; 386)	275 (158; 392)	743 (582; 904)	880 (687; 1,073)	601 (464; 738)	756 (568; 944)	696 (476; 916)	789 (623; 955)	523 (299; 747)	548 (294; 802)	491 (287; 694)	523 (206; 840)	551 (179; 922)	484 (218; 750)
Latvia	307 (115; 499)	565 (313; 816)	117 (-36; 269)	4,060 (3,821; 4,299)	5,488 (5,195; 5,781)	2,896 (2,694; 3,097)	2,267 (1,968; 2,566)	3,513 (3,155; 3,870)	1,201 (941; 1,462)	939 (573; 1,306)	1,962 (1,528; 2,396)	6 (-319; 331)	1,736 (1,257; 2,215)	2,676 (2,090; 3,262)	998 (616; 1,381)
Lithuania	2,194 (1,873; 2,515)	3,305 (2,817; 3,793)	1,368 (1,181; 1,555)	3,942 (3,577; 4,306)	4,702 (4,151; 5,253)	3,189 (2,972; 3,406)	2,107 (1,685; 2,528)	2,358 (1,735; 2,981)	1,798 (1,534; 2,063)	383 (-101; 868)	458 (-248; 1,165)	149 (-165; 463)	2,394 (1,699; 3,089)	3,032 (2,026; 4,038)	1,802 (1,373; 2,231)
Norway	-70 (-219; 80)	-31 (-242; 179)	-97 (-194; 1)	102 (-69; 272)	81 (-153; 315)	121 (6; 236)	826 (631; 1,021)	1,049 (789; 1,309)	627 (489; 765)	627 (406; 849)	787 (498; 1,076)	480 (316; 644)	255 (-58; 568)	352 (-60; 764)	168 (-51; 387)
Sweden	542 (435; 650)	828 (686; 970)	274 (196; 352)	210 (85; 334)	432 (276; 588)	-1 (-99; 97)	286 (136; 436)	451 (274; 628)	121 (-6; 249)	257 (77; 437)	389 (185; 593)	130 (-31; 290)	366 (133; 600)	578 (296; 859)	160 (-25; 344)
**SE**	**898 (752; 1,044)**	**1,072 (889; 1,255)**	**733 (624; 842)**	**1,177 (1,000; 1,355)**	**1,518 (1,296; 1,740)**	**867 (733; 1,000)**	**838 (627; 1,049)**	**994 (732; 1,257)**	**696 (535; 857)**	**287 (39; 536)**	**348 (42; 654)**	**237 (45; 430)**	**878 (785; 970)**	**1,074 (959; 1,190)**	**698 (628; 767)**
Croatia	978 (841; 1,115)	1,157 (984; 1,331)	851 (740; 961)	2,572 (2,408; 2,735)	3,024 (2,824; 3,224)	2,231 (2,093; 2,369)	1,092 (891; 1,293)	1,309 (1,068; 1,551)	906 (732; 1,081)	149 (-93; 391)	316 (28; 604)	36 (-178; 249)	1,341 (1,030; 1,653)	1,601 (1,222; 1,980)	1,146 (896; 1,396)
Cyprus	140 (-20; 301)	101 (-76; 278)	181 (25; 337)	1,216 (986; 1,445)	1,291 (1,021; 1,561)	1,160 (954; 1,366)	724 (424; 1,025)	582 (219; 945)	871 (610; 1,132)	-40 (-413; 333)	-36 (-491; 420)	-37 (-357; 283)	465 (47; 882)	430 (-41; 900)	506 (141; 872)
Greece	436 (329; 544)	492 (376; 607)	385 (280; 490)	1,787 (1,654; 1,920)	2,212 (2,068; 2,357)	1,404 (1,276; 1,532)	985 (825; 1,145)	1,140 (965; 1,315)	884 (732; 1,036)	131 (-58; 320)	131 (-75; 338)	181 (0; 362)	848 (615; 1,081)	1,008 (741; 1,275)	716 (517; 914)

Italy	1,192	1,621	821	1,033	1,328	771	944	1,144	759	595	724	470	1,001	1,280	747
	(1,096; 1,289)	(1,484; 1,759)	(758; 883)	(928; 1,139)	(1,180; 1,476)	(700; 842)	(826; 1,063)	(981; 1,307)	(677; 842)	(461; 728)	(544; 904)	(375; 566)	(818; 1,184)	(1,031; 1,530)	(623; 871)
Malta	445	1,005	-178	536	692	239	479	1,120	-292	-351	38	-860	408	868	-158
	(229; 662)	(784; 1,227)	(-400; 43)	(220; 853)	(363; 1,020)	(-82; 559)	(53; 906)	(671; 1,568)	(-733; 148)	(-892; 189)	(-530; 606)	(-1,416; -304)	(-175; 991)	(263; 1,473)	(-719; 402)
Portugal	696	877	523	768	1,002	559	574	665	467	129	217	21	612	772	456
	(569; 822)	(716; 1,038)	(418; 628)	(624; 913)	(821; 1,183)	(438; 680)	(406; 742)	(457; 873)	(326; 609)	(-65; 324)	(-23; 457)	(-143; 184)	(333; 891)	(429; 1,114)	(232; 679)
Slovenia	1,030	1,261	843	1,148	1,604	762	626	696	560	100	301	-63	878	1,127	678
	(876; 1,184)	(1,054; 1,467)	(730; 957)	(962; 1,335)	(1,362; 1,847)	(621; 904)	(404; 849)	(412; 981)	(387; 732)	(-165; 365)	(-31; 633)	(-275; 149)	(524; 1,233)	(670; 1,584)	(418; 938)
Spain	1,283	1,597	1,013	706	961	487	777	925	640	247	259	229	852	1,061	669
	(1,189; 1,376)	(1,477; 1,718)	(940; 1,085)	(603; 809)	(829; 1,094)	(407; 567)	(660; 894)	(777; 1,073)	(547; 733)	(113; 381)	(91; 427)	(120; 338)	(667; 1,038)	(824; 1,298)	(534; 805)
**WE**	**563**	**766**	**384**	**747**	**1,060**	**460**	**786**	**999**	**584**	**404**	**578**	**242**	**634**	**865**	**423**
	**(414; 713)**	**(570; 963)**	**(285; 484)**	**(570; 925)**	**(832; 1,287)**	**(335; 585)**	**(576; 997)**	**(736; 1,262)**	**(431; 737)**	**(159; 649)**	**(274; 881)**	**(59; 425)**	**(541; 727)**	**(746; 984)**	**(358; 488)**
Austria	645	906	428	932	1,344	560	861	1,165	594	488	795	215	742	1,062	466
	(526; 764)	(743; 1,069)	(346; 509)	(794; 1,071)	(1,160; 1,529)	(459; 660)	(698; 1,024)	(949; 1,380)	(472; 716)	(297; 679)	(545; 1,046)	(70; 361)	(482; 1,002)	(731; 1,393)	(274; 658)
Belgium	1,217	1,554	912	612	977	294	714	860	566	271	348	194	787	1,027	569
	(1,077; 1,358)	(1,361; 1,748)	(815; 1,008)	(458; 765)	(770; 1,183)	(186; 402)	(543; 885)	(635; 1,086)	(442; 690)	(80; 462)	(100; 595)	(53; 336)	(510; 1,063)	(659; 1,395)	(377; 760)
France	571	828	363	697	1,029	414	776	1,026	555	269	391	156	600	851	385
	(483; 659)	(692; 965)	(314; 411)	(598; 796)	(881; 1,178)	(356; 471)	(665; 887)	(863; 1,188)	(487; 623)	(144; 394)	(213; 569)	(76; 236)	(426; 775)	(595; 1,107)	(284; 485)
Germany	179	333	46	670	974	395	897	1,133	688	511	679	360	499	720	302
	(81; 277)	(213; 453)	(-34; 127)	(558; 782)	(840; 1,107)	(300; 491)	(767; 1,028)	(981; 1,285)	(574; 801)	(359; 663)	(505; 854)	(225; 495)	(312; 687)	(502; 939)	(145; 459)
Luxembourg	270	302	263	-50	-248	152	-337	-499	-224	-642	-1,368	30	56	-42	162
	(90; 450)	(74; 531)	(119; 408)	(-325; 225)	(-593; 97)	(-75; 378)	(-714; 39)	(-971; -28)	(-537; 90)	(-1,124; -160)	(-1,973; -762)	(-372; 433)	(-447; 558)	(-648; 564)	(-242; 565)
Netherlands	726	1,044	444	989	1,269	744	785	928	644	624	823	428	797	1,048	559
	(621; 831)	(895; 1,193)	(369; 518)	(871; 1,106)	(1,110; 1,428)	(654; 833)	(648; 923)	(750; 1,106)	(532; 756)	(466; 783)	(624; 1,022)	(293; 562)	(586; 1,007)	(790; 1,305)	(396; 723)
**Total**	**859**	**1,193**	**590**	**1,581**	**2,123**	**1,119**	**866**	**1,090**	**671**	**254**	**352**	**168**	**974**	**1,304**	**695**
	**(776; 942)**	**(1,089; 1,297)**	**(531; 649)**	**(1,482; 1,681)**	**(1,999; 2,248)**	**(1,045; 1,193)**	**(748; 985)**	**(943; 1,236)**	**(581; 761)**	**(114; 393)**	**(182; 522)**	**(60; 276)**	**(922; 1,026)**	**(1,239; 1,369)**	**(657; 734)**

Notes: Each cell contains a point estimate and a 95% confidence interval in brackets. CEE – Central and Eastern Europe; NE – Northern Europe; SE – Southern Europe; WE – Western Europe.

Table S3. Age-standardised excess years of potential productive life lost per 100,000 working population in 28 countries in 2020-2023

	2020			2021			2022			2023			2020-2023		
Country	Total	Males	Females	Total	Males	Females	Total	Males	Females	Total	Males	Females	Total	Males	Females
**CEE**	**351 (295; 407)**	**509 (428; 590)**	**187 (158; 217)**	**997 (923; 1,070)**	**1,383 (1,278; 1,488)**	**575 (536; 615)**	**224 (133; 315)**	**315 (186; 443)**	**135 (85; 184)**	**-51 (-160; 58)**	**-59 (-212; 94)**	**-21 (-81; 39)**	**451 (413; 489)**	**636 (582; 690)**	**256 (236; 276)**
Bulgaria	653 (614; 691)	940 (885; 994)	363 (340; 387)	1,767 (1,707; 1,826)	2,337 (2,254; 2,419)	1,190 (1,151; 1,229)	173 (91; 256)	148 (34; 262)	204 (148; 259)	-329 (-436; -222)	-460 (-608; -311)	-195 (-267; -122)	783 (704; 863)	1,068 (959; 1,176)	491 (442; 540)
Czechia	124 (79; 169)	154 (88; 220)	94 (69; 118)	524 (469; 578)	709 (631; 787)	332 (301; 364)	213 (147; 279)	329 (236; 422)	89 (49; 129)	82 (5; 160)	201 (93; 309)	-41 (-91; 8)	243 (169; 318)	346 (241; 451)	141 (99; 184)
Hungary	248 (119; 377)	324 (133; 515)	173 (104; 241)	1,140 (997; 1,283)	1,509 (1,297; 1,721)	766 (690; 843)	290 (131; 449)	410 (177; 644)	167 (79; 255)	81 (-96; 257)	43 (-216; 302)	116 (15; 217)	471 (300; 643)	613 (368; 859)	326 (230; 423)
Poland	413 (348; 478)	663 (559; 767)	168 (141; 195)	887 (813; 960)	1,364 (1,246; 1,481)	416 (385; 448)	383 (301; 466)	562 (432; 692)	206 (170; 242)	9 (-85; 103)	-47 (-194; 101)	65 (24; 106)	473 (376; 570)	719 (563; 876)	228 (190; 266)
Romania	387 (336; 438)	505 (433; 577)	266 (236; 296)	987 (921; 1,054)	1,267 (1,173; 1,361)	698 (660; 736)	104 (21; 188)	141 (23; 258)	66 (18; 115)	-227 (-330; -125)	-338 (-482; -194)	-118 (-179; -58)	425 (337; 514)	542 (417; 668)	307 (256; 357)
Slovakia	39 (-19; 98)	73 (-19; 165)	6 (-24; 35)	870 (800; 941)	1,292 (1,186; 1,398)	444 (401; 486)	225 (138; 312)	425 (301; 549)	19 (-40; 77)	41 (-63; 146)	117 (-28; 261)	-41 (-115; 34)	299 (198; 401)	482 (337; 626)	117 (59; 174)
**NE**	**51 (-11; 113)**	**83 (4; 163)**	**-1 (-42; 40)**	**124 (46; 202)**	**165 (68; 261)**	**79 (23; 135)**	**140 (43; 238)**	**214 (97; 331)**	**63 (-10; 135)**	**145 (27; 263)**	**226 (87; 365)**	**42 (-48; 132)**	**98 (57; 139)**	**149 (98; 200)**	**34 (5; 62)**
Denmark	50 (-26; 126)	64 (-32; 160)	35 (-22; 92)	35 (-53; 124)	3 (-105; 112)	67 (-1; 135)	140 (36; 243)	222 (96; 347)	56 (-27; 139)	112 (-9; 234)	174 (31; 318)	49 (-51; 149)	71 (-52; 195)	95 (-55; 244)	49 (-48; 146)
Estonia	244 (83; 404)	514 (261; 767)	-53 (-118; 13)	776 (583; 968)	1,242 (959; 1,526)	282 (182; 382)	867 (635; 1,098)	1,513 (1,188; 1,839)	172 (35; 308)	761 (489; 1,032)	1,378 (1,006; 1,749)	101 (-74; 275)	578 (295; 861)	1,060 (645; 1,475)	71 (-67; 208)
Finland	117 (55; 178)	271 (176; 365)	-43 (-73; -12)	-13 (-89; 63)	24 (-89; 136)	-48 (-89; -8)	110 (17; 204)	175 (41; 309)	46 (-8; 99)	119 (5; 232)	247 (87; 407)	-14 (-82; 55)	81 (-29; 191)	185 (24; 345)	-28 (-85; 29)
Ireland	110 (57; 163)	175 (99; 251)	47 (12; 81)	200 (134; 266)	310 (226; 394)	90 (37; 143)	143 (61; 226)	200 (104; 296)	88 (14; 163)	129 (27; 230)	253 (144; 362)	5 (-94; 104)	142 (47; 238)	230 (114; 346)	59 (-13; 131)
Latvia	-71 (-188; 46)	-31 (-211; 149)	-102 (-167; -38)	1,546 (1,390; 1,702)	2,467 (2,240; 2,695)	676 (582; 769)	1,188 (980; 1,395)	2,104 (1,806; 2,401)	299 (172; 426)	685 (422; 949)	1,525 (1,148; 1,902)	-133 (-295; 29)	610 (382; 839)	1,127 (800; 1,454)	141 (7; 274)
Lithuania	787 (549; 1,026)	1,314 (922; 1,705)	302 (207; 398)	1,510 (1,246; 1,774)	2,200 (1,772; 2,627)	867 (758; 977)	860 (562; 1,158)	1,143 (668; 1,618)	603 (473; 733)	508 (171; 846)	789 (256; 1,321)	252 (100; 405)	956 (566; 1,347)	1,427 (794; 2,059)	513 (356; 669)
Norway	20 (-35; 75)	0 (-68; 69)	41 (-1; 83)	-31 (-99; 36)	-85 (-167; -3)	26 (-29; 81)	150 (67; 233)	169 (72; 266)	131 (58; 204)	222 (122; 322)	254 (140; 367)	190 (97; 283)	54 (-48; 155)	42 (-80; 165)	67 (-12; 146)
Sweden	-7 (-48; 34)	16 (-34; 66)	-29 (-62; 4)	28 (-20; 77)	67 (9; 125)	-9 (-50; 31)	-12 (-70; 47)	68 (-1; 138)	-97 (-147; -46)	39 (-32; 110)	68 (-16; 152)	11 (-51; 72)	7 (-62; 77)	45 (-37; 127)	-31 (-87; 26)
**SE**	**106 (65; 147)**	**148 (91; 205)**	**66 (42; 90)**	**234 (184; 285)**	**340 (270; 409)**	**133 (102; 164)**	**137 (77; 198)**	**197 (116; 279)**	**82 (43; 120)**	**84 (12; 155)**	**133 (39; 227)**	**40 (-7; 86)**	**143 (117; 169)**	**206 (170; 242)**	**83 (67; 99)**
Croatia	39 (-12; 90)	24 (-48; 95)	57 (24; 89)	449 (384; 514)	550 (464; 636)	357 (310; 405)	116 (32; 200)	159 (50; 268)	76 (11; 140)	-5 (-110; 99)	-41 (-176; 94)	33 (-49; 115)	156 (65; 247)	185 (60; 309)	132 (74; 189)
Cyprus	162 (102; 221)	174 (102; 246)	160 (108; 213)	449 (356; 542)	616 (497; 736)	298 (222; 373)	122 (-7; 251)	138 (-31; 307)	128 (24; 231)	154 (-11; 320)	275 (57; 492)	62 (-74; 198)	224 (95; 353)	275 (116; 435)	182 (81; 283)
Greece	60 (23; 97)	28 (-24; 80)	90 (68; 112)	420 (372; 468)	601 (531; 671)	247 (220; 274)	101 (40; 162)	118 (29; 206)	83 (49; 118)	-40 (-115; 34)	-128 (-236; -19)	45 (2; 87)	150 (83; 216)	172 (79; 265)	127 (87; 167)

Italy	89 (64; 114)	135 (99; 170)	46 (30; 61)	170 (141; 198)	247 (208; 286)	94 (74; 113)	135 (102; 168)	196 (152; 239)	75 (50; 99)	129 (90; 167)	198 (150; 246)	59 (29; 90)	127 (85; 169)	190 (134; 246)	65 (38; 92)
Malta	155 (71; 240)	424 (328; 520)	-151 (-226; -76)	86 (-53; 225)	383 (218; 547)	-253 (-371; -136)	211 (14; 408)	565 (328; 802)	-210 (-373; -46)	-106 (-367; 155)	129 (-187; 446)	-379 (-592; -165)	129 (-51; 309)	412 (207; 617)	-199 (-349; -49)
Portugal	185 (125; 245)	356 (258; 454)	23 (-4; 51)	171 (99; 243)	299 (189; 410)	53 (11; 95)	149 (63; 236)	318 (192; 444)	-12 (-69; 46)	91 (-11; 194)	245 (102; 389)	-59 (-133; 15)	163 (61; 265)	316 (166; 465)	19 (-37; 76)
Slovenia	97 (31; 162)	92 (-1; 185)	107 (68; 147)	175 (87; 262)	249 (131; 367)	101 (44; 157)	194 (82; 305)	168 (22; 313)	226 (150; 302)	98 (-39; 235)	52 (-124; 229)	159 (63; 255)	132 (9; 255)	141 (-24; 306)	126 (50; 202)
Spain	171 (129; 213)	241 (179; 302)	101 (78; 124)	146 (100; 192)	212 (144; 280)	79 (54; 103)	155 (104; 205)	211 (136; 286)	97 (70; 124)	85 (29; 141)	137 (54; 220)	32 (2; 61)	146 (87; 205)	209 (124; 294)	82 (50; 115)
**WE**	**56 (11; 101)**	**105 (47; 163)**	**6 (-20; 33)**	**160 (106; 214)**	**236 (168; 304)**	**55 (21; 88)**	**170 (107; 233)**	**243 (164; 323)**	**78 (37; 119)**	**117 (44; 191)**	**187 (95; 280)**	**36 (-14; 85)**	**116 (88; 144)**	**181 (145; 216)**	**36 (18; 53)**
Austria	14 (-30; 59)	57 (-11; 124)	-28 (-51; -4)	167 (113; 221)	338 (262; 414)	-7 (-43; 29)	129 (64; 195)	258 (171; 345)	0 (-49; 49)	108 (30; 186)	212 (112; 312)	6 (-57; 69)	87 (11; 162)	196 (93; 299)	-16 (-62; 30)
Belgium	107 (55; 159)	175 (107; 243)	38 (1; 76)	161 (102; 221)	196 (119; 273)	127 (84; 170)	156 (88; 224)	214 (126; 301)	97 (47; 147)	93 (16; 170)	109 (10; 207)	77 (20; 135)	130 (47; 212)	178 (73; 283)	80 (21; 139)
France	45 (7; 83)	82 (23; 142)	10 (-8; 27)	101 (57; 144)	195 (128; 262)	9 (-12; 30)	148 (99; 197)	249 (174; 323)	50 (24; 76)	92 (36; 148)	177 (94; 260)	8 (-23; 40)	88 (29; 146)	163 (74; 252)	16 (-12; 44)
Germany	54 (14; 93)	104 (49; 160)	2 (-22; 26)	189 (146; 233)	301 (240; 361)	76 (50; 102)	213 (164; 261)	317 (250; 384)	106 (76; 135)	144 (90; 198)	241 (166; 316)	45 (11; 79)	140 (83; 196)	227 (149; 305)	50 (17; 83)
Luxembourg	-33 (-102; 36)	-83 (-163; -3)	19 (-43; 80)	3 (-106; 112)	-123 (-248; 2)	134 (38; 230)	-71 (-224; 82)	-121 (-300; 58)	-23 (-156; 110)	-96 (-294; 102)	-158 (-392; 76)	-30 (-201; 141)	-34 (-176; 108)	-103 (-265; 60)	37 (-83; 158)
Netherlands	95 (55; 135)	147 (106; 189)	43 (3; 82)	206 (162; 250)	237 (192; 283)	174 (130; 218)	198 (149; 248)	228 (176; 280)	168 (119; 218)	149 (92; 205)	200 (142; 259)	96 (39; 153)	154 (95; 213)	195 (135; 256)	112 (55; 169)
**Total**	**129 (104; 153)**	**182 (149; 215)**	**69 (55; 83)**	**324 (294; 354)**	**427 (387; 467)**	**201 (182; 220)**	**163 (126; 199)**	**233 (185; 280)**	**90 (67; 113)**	**82 (39; 126)**	**142 (87; 198)**	**26 (-2; 54)**	**181 (166; 197)**	**252 (231; 273)**	**102 (93; 112)**

Notes: Each cell contains a point estimate and a 95% confidence interval in brackets. CEE – Central and Eastern Europe; NE – Northern Europe; SE – Southern Europe; WE – Western Europe.

Table S4. Excess deaths in 5-year age groups in 28 European countries in 2020-2023

	2020		2021		2022		2023		
Age group	Males	Females	Males	Females	Males	Females	Males	Females	Total
0-4	-749<br>(-1,253; -246)	-368<br>(-770; 34)	-540<br>(-1,160; 81)	-181<br>(-680; 318)	-139<br>(-888; 611)	-36<br>(-647; 574)	-270<br>(-1,159; 619)	-86<br>(-813; 641)	-2,369<br>(-7,369; 2,631)
5-9	-25<br>(-126; 75)	-85<br>(-162; -9)	12<br>(-141; 164)	18<br>(-86; 123)	66<br>(-145; 278)	138<br>(2; 274)	74<br>(-199; 347)	134<br>(-44; 311)	331<br>(-901; 1,563)
10-14	-73<br>(-178; 32)	-61<br>(-142; 19)	-26<br>(-184; 131)	28<br>(-101; 156)	95<br>(-117; 307)	67<br>(-107; 240)	78<br>(-198; 353)	59<br>(-170; 287)	165<br>(-1,197; 1,527)
15-19	-118<br>(-364; 129)	-88<br>(-186; 9)	149<br>(-172; 470)	11<br>(-126; 147)	167<br>(-239; 574)	175<br>(-15; 366)	105<br>(-400; 611)	145<br>(-104; 394)	546<br>(-1,607; 2,700)
20-24	174<br>(-250; 598)	-46<br>(-203; 110)	482<br>(-42; 1,007)	142<br>(-83; 367)	657<br>(38; 1,276)	303<br>(4; 603)	453<br>(-288; 1,194)	165<br>(-219; 550)	2,331<br>(-1,044; 5,705)
25-29	25<br>(-399; 450)	-21<br>(-159; 117)	517<br>(-22; 1,056)	248<br>(61; 435)	344<br>(-327; 1,014)	178<br>(-63; 419)	292<br>(-524; 1,109)	71<br>(-230; 373)	1,655<br>(-1,663; 4,972)
30-34	278<br>(-180; 737)	119<br>(-68; 306)	1,060<br>(468; 1,653)	525<br>(232; 817)	666<br>(-90; 1,422)	312<br>(-90; 714)	147<br>(-789; 1,084)	-87<br>(-608; 433)	3,021<br>(-1,124; 7,165)
35-39	1,025<br>(439; 1,610)	295<br>(24; 566)	2,441<br>(1,693; 3,190)	851<br>(482; 1,220)	1,870<br>(926; 2,813)	495<br>(11; 978)	1,157<br>(-13; 2,327)	353<br>(-260; 965)	8,486<br>(3,303; 13,669)
40-44	2,092<br>(733; 3,451)	916<br>(350; 1,481)	4,630<br>(3,048; 6,212)	1,982<br>(1,291; 2,672)	3,174<br>(1,385; 4,962)	1,178<br>(335; 2,021)	2,337<br>(264; 4,411)	607<br>(-418; 1,631)	16,915<br>(6,988; 26,841)
45-49	3,343<br>(679; 6,006)	1,389<br>(263; 2,515)	7,546<br>(4,564; 10,528)	3,253<br>(2,010; 4,497)	3,668<br>(337; 6,999)	1,541<br>(143; 2,939)	2,071<br>(-1,679; 5,821)	729<br>(-857; 2,315)	23,540<br>(5,459; 41,620)
50-54	5,849<br>(1,408; 10,290)	1,880<br>(110; 3,651)	12,204<br>(7,278; 17,130)	4,758<br>(2,694; 6,822)	6,342<br>(816; 11,868)	1,691<br>(-732; 4,115)	2,303<br>(-3,809; 8,415)	14<br>(-2,780; 2,809)	35,042<br>(4,986; 65,099)
55-59	7,769<br>(2,748; 12,790)	2,092<br>(556; 3,627)	15,926<br>(10,443; 21,410)	6,019<br>(4,139; 7,899)	6,861<br>(755; 12,966)	1,293<br>(-1,023; 3,610)	950<br>(-5,927; 7,826)	-1,187<br>(-4,009; 1,635)	39,723<br>(7,681; 71,764)
60-64	13,110<br>(8,550; 17,670)	4,401<br>(2,789; 6,012)	24,516<br>(19,334; 29,699)	12,521<br>(10,472; 14,571)	7,169<br>(1,090; 13,248)	2,932<br>(366; 5,497)	-3,645<br>(-10,721; 3,430)	-2,467<br>(-5,616; 682)	58,536<br>(26,263; 90,809)
65-69	24,240<br>(18,640; 29,839)	10,082<br>(7,550; 12,614)	40,752<br>(34,222; 47,281)	24,299<br>(21,178; 27,421)	17,257<br>(9,510; 25,003)	10,931<br>(7,043; 14,819)	3,419<br>(-5,663; 12,500)	4,070<br>(-625; 8,765)	135,050<br>(91,856; 178,243)
70-74	33,255<br>(27,763; 38,747)	16,535<br>(13,135; 19,936)	46,297<br>(39,383; 53,212)	29,884<br>(25,328; 34,440)	20,897<br>(12,386; 29,407)	11,631<br>(5,794; 17,468)	1,903<br>(-8,426; 12,232)	-194<br>(-7,340; 6,952)	160,209<br>(108,024; 212,393)
75-79	37,790<br>(25,827; 49,752)	25,507<br>(17,109; 33,905)	55,595<br>(42,877; 68,314)	44,363<br>(35,247; 53,480)	40,791<br>(26,419; 55,162)	33,185<br>(22,757; 43,613)	23,056<br>(6,476; 39,635)	18,454<br>(6,251; 30,657)	278,740<br>(182,962; 374,518)
80-84	49,260<br>(33,646; 64,874)	38,696<br>(23,920; 53,472)	47,373<br>(30,133; 64,612)	46,488<br>(30,473; 62,504)	37,492<br>(18,327; 56,657)	36,483<br>(19,060; 53,905)	17,173<br>(-4,248; 38,595)	15,034<br>(-4,141; 34,208)	287,998<br>(147,170; 428,826)
85+	71,798<br>(62,028; 81,568)	101,585<br>(87,466; 115,704)	56,791<br>(44,092; 69,491)	71,863<br>(51,205; 92,521)	69,005<br>(52,964; 85,047)	102,718<br>(74,023; 131,414)	11,697<br>(-8,409; 31,802)	4,659<br>(-32,369; 41,688)	490,116<br>(330,999; 649,234)
Total	249,043<br>(179,713; 318,374)	202,826<br>(151,584; 254,068)	315,726<br>(235,811; 395,642)	247,072<br>(183,735; 310,409)	216,381<br>(123,148; 309,614)	205,214<br>(126,861; 283,568)	63,299<br>(-45,712; 172,310)	40,472<br>(-54,352; 135,295)	1,540,034<br>(900,788; 2,179,279)

Notes: Each cell contains a point estimate and a 95% confidence interval in parentheses.

Table S5. Share of excess deaths in selected age-specific sub-populations in 28 European countries in 2020-2023

	2020		2021		2022		2023		2020-2023		
Sub-population	Males	Females	Males	Females	Males	Females	Males	Females	Males	Females	Total
aged <65	13.1%	5.1%	21.8%	12.2%	14.3%	5.0%	9.6%	-3.8%	16.4%	7.1%	12.2%
aged 85+	28.8%	50.1%	18.0%	29.1%	31.9%	50.1%	18.5%	11.5%	24.8%	40.4%	31.8%

Notes: The shares refer to point estimates as reported in Table S4.

Table S6. Excess years of life lost (eYLL) in 5-year age groups in 28 European countries in 2020-2023

	2020		2021		2022		2023		2020-2023
Age group	Males	Females	Males	Females	Males	Females	Males	Females	Total
0-4	-57,475<br>(-94,885; -20,065)	-30,233<br>(-62,666; 2,200)	-41,959<br>(-88,249; 4,331)	-14,864<br>(-55,243; 25,514)	-11,691<br>(-67,777; 44,396)	-2,712<br>(-52,172; 46,748)	-21,282<br>(-87,936; 45,371)	-6,495<br>(-65,502; 52,512)	-186,711<br>(-574,429; 201,006)
5-9	-1,978<br>(-9,063; 5,107)	-6,449<br>(-12,273; -624)	834<br>(-9,959; 11,628)	1,451<br>(-6,555; 9,457)	4,968<br>(-10,010; 19,945)	10,708<br>(336; 21,080)	5,491<br>(-13,872; 24,855)	10,486<br>(-3,088; 24,061)	25,512<br>(-64,486; 115,510)
10-14	-4,644<br>(-11,475; 2,187)	-4,324<br>(-10,084; 1,435)	-1,765<br>(-12,115; 8,585)	2,075<br>(-7,099; 11,248)	6,370<br>(-7,574; 20,313)	4,865<br>(-7,504; 17,235)	5,044<br>(-13,114; 23,203)	4,344<br>(-11,988; 20,676)	11,965<br>(-80,952; 104,883)
15-19	-7,419<br>(-22,431; 7,592)	-5,654<br>(-12,195; 887)	9,012<br>(-10,548; 28,573)	819<br>(-8,307; 9,945)	11,033<br>(-13,732; 35,799)	12,046<br>(-699; 24,792)	7,201<br>(-23,614; 38,015)	10,052<br>(-6,614; 26,717)	37,090<br>(-98,140; 172,320)
20-24	9,142<br>(-14,663; 32,947)	-2,933<br>(-12,575; 6,708)	26,239<br>(-3,188; 55,665)	8,463<br>(-5,419; 22,345)	37,553<br>(2,860; 72,247)	18,776<br>(293; 37,260)	26,310<br>(-15,191; 67,811)	10,519<br>(-13,169; 34,207)	134,069<br>(-61,053; 329,191)
25-29	1,136<br>(-20,847; 23,119)	-1,391<br>(-9,230; 6,448)	25,581<br>(-2,443; 53,606)	13,487<br>(2,847; 24,127)	17,933<br>(-16,992; 52,858)	9,978<br>(-3,710; 23,667)	15,533<br>(-27,112; 58,177)	3,959<br>(-13,184; 21,101)	86,216<br>(-90,671; 263,102)
30-34	12,501<br>(-8,525; 33,527)	5,923<br>(-3,777; 15,623)	46,753<br>(19,592; 73,913)	26,041<br>(10,826; 41,255)	30,745<br>(-3,886; 65,375)	16,104<br>(-4,827; 37,034)	8,065<br>(-34,853; 50,984)	-4,540<br>(-31,667; 22,587)	141,591<br>(-57,117; 340,300)
35-39	41,077<br>(17,131; 65,023)	13,342<br>(543; 26,141)	96,866<br>(66,181; 127,551)	38,717<br>(21,286; 56,148)	75,907<br>(37,087; 114,728)	22,991<br>(145; 45,837)	48,601<br>(459; 96,742)	16,461<br>(-12,469; 45,391)	353,961<br>(130,362; 577,561)
40-44	75,837<br>(26,003; 125,672)	38,139<br>(14,231; 62,047)	163,311<br>(105,395; 221,228)	80,913<br>(51,704; 110,122)	118,890<br>(53,412; 184,368)	49,621<br>(13,915; 85,327)	88,999<br>(13,234; 164,764)	26,211<br>(-17,187; 69,609)	641,922<br>(260,706; 1,023,138)
45-49	105,016<br>(18,770; 191,261)	50,826<br>(8,714; 92,938)	231,316<br>(135,026; 327,606)	116,149<br>(69,710; 162,588)	117,260<br>(10,060; 224,460)	56,898<br>(4,767; 109,030)	70,861<br>(-49,507; 191,228)	27,596<br>(-31,471; 86,664)	775,923<br>(166,069; 1,385,777)
50-54	159,922<br>(34,852; 284,993)	59,832<br>(1,857; 117,808)	323,284<br>(185,179; 461,390)	148,579<br>(81,191; 215,967)	178,413<br>(24,114; 332,711)	55,988<br>(-22,906; 134,881)	73,341<br>(-96,837; 243,519)	2,392<br>(-88,461; 93,245)	1,001,752<br>(118,989; 1,884,515)
55-59	179,807<br>(59,068; 300,545)	56,392<br>(13,287; 99,497)	361,108<br>(229,421; 492,796)	158,112<br>(105,232; 210,992)	164,481<br>(17,893; 311,069)	35,835<br>(-29,469; 101,138)	31,205<br>(-133,704; 196,115)	-32,199<br>(-111,831; 47,433)	954,741<br>(149,897; 1,759,585)
60-64	252,833<br>(163,986; 341,679)	99,997<br>(61,915; 138,079)	457,036<br>(355,848; 558,224)	278,194<br>(229,727; 326,661)	143,607<br>(24,568; 262,646)	69,857<br>(9,182; 130,532)	-63,801<br>(-202,813; 75,212)	-54,856<br>(-129,424; 19,712)	1,182,867<br>(512,990; 1,852,744)
65-69	384,848<br>(292,713; 476,984)	190,115<br>(141,168; 239,062)	628,916<br>(522,019; 735,814)	449,042<br>(388,698; 509,386)	284,627<br>(158,230; 411,023)	216,025<br>(140,956; 291,094)	69,156<br>(-78,827; 217,139)	86,109<br>(-4,458; 176,675)	2,308,838<br>(1,560,499; 3,057,178)
70-74	424,843<br>(352,089; 497,597)	249,880<br>(197,689; 302,071)	566,653<br>(475,853; 657,453)	431,453<br>(361,494; 501,412)	270,244<br>(159,207; 381,281)	180,228<br>(90,568; 269,888)	33,161<br>(-101,090; 167,413)	1,147<br>(-108,682; 110,975)	2,157,609<br>(1,427,128; 2,888,089)
75-79	374,461<br>(253,614; 495,309)	298,403<br>(200,099; 396,706)	531,633<br>(403,036; 660,231)	498,337<br>(391,668; 605,006)	407,529<br>(262,127; 552,931)	394,682<br>(272,619; 516,744)	235,657<br>(67,984; 403,329)	226,808<br>(83,849; 369,767)	2,967,510<br>(1,934,996; 4,000,024)
80-84	356,679<br>(242,128; 471,230)	332,953<br>(207,151; 458,754)	329,508<br>(203,203; 455,813)	373,080<br>(236,686; 509,473)	270,369<br>(130,243; 410,494)	312,322<br>(163,979; 460,665)	128,094<br>(-28,274; 284,463)	137,146<br>(-26,069; 300,361)	2,240,150<br>(1,129,047; 3,351,253)
85+	253,798<br>(219,622; 287,975)	416,900<br>(361,055; 472,745)	196,646<br>(152,228; 241,065)	275,988<br>(193,929; 358,046)	241,247<br>(185,026; 297,468)	411,856<br>(297,320; 526,392)	41,722<br>(-28,760; 112,203)	22,203<br>(-125,726; 170,131)	1,860,360<br>(1,254,694; 2,466,025)
Total	2,560,384<br>(1,498,088; 3,622,680)	1,761,718<br>(1,084,910; 2,438,526)	3,950,974<br>(2,726,476; 5,175,472)	2,886,035<br>(2,062,376; 3,709,694)	2,369,484<br>(944,856; 3,794,113)	1,876,068<br>(872,791; 2,879,345)	803,357<br>(-853,828; 2,460,542)	487,343<br>(-717,140; 1,691,827)	16,695,365<br>(7,618,529; 25,772,200)

Notes: Each cell contains a point estimate and a 95% confidence interval in parentheses.

Table S7. Share of excess years of life lost (eYLL) in selected age-specific sub-populations in 28 European countries in 2020-2023

	2020		2021		2022		2023		2020-2023		
Sub-population	Males	Females	Males	Females	Males	Females	Males	Females	Males	Females	Total
aged <60	20.0%	9.8%	31.4%	20.1%	31.7%	15.5%	44.7%	14.1%	29.6%	15.9%	23.8%
aged 75+	38.5%	59.5%	26.8%	39.8%	38.8%	59.6%	50.5%	79.2%	34.8%	52.8%	42.3%

Notes: The shares refer to point estimates as reported in Table S6.

Table S8. Excess years of potential productive life lost (eYPPLL) in 5-year age groups in 28 European countries in 2020-2023

	2020		2021		2022		2023		2020-2023
Age group	Males	Females	Males	Females	Males	Females	Males	Females	Total
20-24	7,377<br>(-10,335; 25,090)	-2,027<br>(-8,507; 4,454)	20,103<br>(-1,814; 42,019)	5,719<br>(-3,615; 15,052)	27,356<br>(1,515; 53,197)	12,318<br>(-111; 24,747)	18,827<br>(-12,081; 49,735)	6,750<br>(-9,184; 22,684)	96,423<br>(-44,132; 236,978)
25-29	1,078<br>(-14,498; 16,653)	-827<br>(-5,856; 4,202)	19,122<br>(-662; 38,906)	8,780<br>(1,960; 15,601)	12,650<br>(-11,937; 37,237)	6,316<br>(-2,452; 15,084)	10,796<br>(-19,173; 40,765)	2,477<br>(-8,500; 13,453)	60,391<br>(-61,119; 181,901)
30-34	8,858<br>(-5,760; 23,476)	3,514<br>(-2,352; 9,380)	33,713<br>(14,859; 52,566)	15,890<br>(6,690; 25,089)	21,389<br>(-2,652; 45,430)	9,541<br>(-3,114; 22,197)	4,841<br>(-24,939; 34,620)	-2,879<br>(-19,278; 13,521)	94,866<br>(-36,546; 226,279)
35-39	27,868<br>(12,169; 43,568)	7,538<br>(406; 14,670)	65,755<br>(45,703; 85,808)	21,885<br>(12,185; 31,585)	50,350<br>(25,064; 75,635)	12,728<br>(16; 25,440)	31,214<br>(-119; 62,547)	9,186<br>(-6,922; 25,295)	226,524<br>(88,501; 364,548)
40-44	45,752<br>(16,099; 75,406)	19,320<br>(7,320; 31,320)	100,892<br>(66,383; 135,400)	41,374<br>(26,746; 56,003)	69,027<br>(30,036; 108,017)	25,181<br>(7,326; 43,036)	50,722<br>(5,507; 95,936)	13,197<br>(-8,481; 34,875)	365,464<br>(150,935; 579,993)
45-49	56,341<br>(11,594; 101,088)	22,785<br>(4,278; 41,293)	127,090<br>(77,006; 177,174)	51,858<br>(31,471; 72,246)	62,004<br>(6,017; 117,991)	25,623<br>(2,745; 48,502)	35,332<br>(-27,707; 98,372)	12,974<br>(-12,948; 38,896)	394,008<br>(92,454; 695,563)
50-54	69,546<br>(16,990; 122,102)	20,436<br>(227; 40,645)	144,028<br>(85,853; 202,202)	51,760<br>(28,267; 75,253)	75,685<br>(10,590; 140,780)	19,872<br>(-7,639; 47,384)	28,088<br>(-43,819; 99,996)	1,253<br>(-30,420; 32,925)	410,668<br>(60,049; 761,288)
55-59	52,358<br>(18,443; 86,273)	11,768<br>(2,015; 21,521)	108,139<br>(71,131; 145,146)	34,866<br>(22,975; 46,758)	46,557<br>(5,339; 87,774)	8,392<br>(-6,244; 23,027)	5,962<br>(-40,510; 52,435)	-6,375<br>(-24,194; 11,444)	261,667<br>(48,956; 474,378)
60-64	22,628<br>(15,022; 30,233)	4,040<br>(2,026; 6,055)	40,442<br>(31,675; 49,208)	8,829<br>(6,166; 11,491)	13,015<br>(2,651; 23,380)	4,164<br>(754; 7,574)	-4,975<br>(-17,099; 7,150)	-1,060<br>(-5,350; 3,230)	87,083<br>(35,845; 138,321)
Total	291,806<br>(59,723; 523,889)	86,548<br>(-443; 173,539)	659,282<br>(390,134; 928,430)	240,961<br>(132,844; 349,078)	378,032<br>(66,622; 689,443)	124,135<br>(-8,720; 256,990)	180,808<br>(-179,940; 541,556)	35,523<br>(-125,277; 196,323)	1,997,095<br>(334,943; 3,659,247)

Notes: Each cell contains a point estimate and a 95% confidence interval in parentheses.

Table S9. Share of excess years of potential life lost (eYPPLL) in selected age-specific sub-populations in 28 European countries in 2020-2023

	2020		2021		2022		2023		2020-2023		
Sub-population	Males	Females	Males	Females	Males	Females	Males	Females	Males	Females	Total
aged <45	31.2%	31.8%	36.3%	38.9%	47.8%	53.2%	64.4%	80.9%	41.6%	44.3%	42.2%
aged 45+	68.8%	68.2%	63.7%	61.1%	52.2%	46.8%	35.6%	19.1%	58.4%	55.7%	57.8%

Notes: The shares refer to point estimates as reported in Table S8.

Table S10. Sensitivity analysis: Absolute percentage deviations from baseline model (S2), by age group and model specification

Age-group	S1: long_OLS	S3: long_sq	S4: short_sq	ARIMA	ETS
**Median absolute deviation**					
0-24	8.6%	10.4%	23.1%	7.7%	12.7%
25-64	3.4%	3.7%	7.2%	3.6%	4.1%
65+	1.9%	1.9%	3.1%	1.8%	2.2%
total	3.5%	3.9%	7.4%	3.5%	4.5%
**1st quartile**					
0-24	4.4%	4.5%	8.4%	3.3%	6.4%
25-64	1.5%	1.3%	2.6%	1.5%	1.8%
65+	1.0%	0.8%	1.3%	0.8%	0.9%
total	1.5%	1.4%	2.6%	1.4%	1.8%
**3rd quartile**					
0-24	15.4%	24.3%	53.8%	18.9%	26.9%
25-64	6.5%	9.3%	17.5%	7.9%	8.8%
65+	3.3%	3.8%	6.4%	3.6%	4.1%
total	7.6%	10.4%	21.0%	8.5%	11.1%
**Interquartile range**					
0-24	11.0%	19.8%	45.4%	15.6%	20.6%
25-64	5.1%	8.0%	14.9%	6.5%	7.1%
65+	2.4%	3.0%	5.1%	2.8%	3.1%
total	6.1%	9.0%	18.4%	7.1%	9.3%
**Minimum value**					
0-24	0.02%	0.07%	0.02%	<0.01%	0.05%
25-64	<0.01%	<0.01%	0.01%	<0.01%	0.02%
65+	0.01%	<0.01%	0.01%	<0.01%	<0.01%
total	<0.01%	<0.01%	0.01%	<0.01%	<0.01%
**Maximum value**					
0-24	2,231.5%	11,483.7%	4,097.3%	5,925.9%	7,335.0%
25-64	311.5%	313.4%	1477.4%	2,066.1%	316.9%
65+	13.4%	31.7%	63.4%	2,438.2%	24.7%
total	2,231.5%	11,483.7%	4,097.3%	5,925.9%	7,335.0%

Notes: Values represent deviations of each alternative model from the baseline (S2), summarised as median of absolute deviations, 1st and 3rd quartiles, interquartile range, and extreme values. Model description: S1 – linear trend with full baseline period 2002-2019; S2 – linear trend with shorter baseline period 2010-2019; S3 – linear and quadratic trends with full baseline period 2002-2019; S4 – linear and quadratic trends with shorter baseline period 2010-2019; ARIMA – Autoregressive Moving Average model based on full baseline period 2002-2019; ETS – exponential smoothing model based on full baseline period 2002-2019. For more details on models' estimation procedure, see Methods section of the paper.

Table S11. Sensitivity analysis: excess Years of Life Lost (eYLL) and excess Years of Potential Productive Life Lost (eYLL) under alternative assumptions on life expectancy and productive ages

	eYLL		eYPPLL	
**Country**	**Base scenario**	**LE_2050**	**Base scenario**	**20-64**
**CEE**	**5,775,013**	**29.7%**	**818,288**	**5.9%**
Bulgaria	704,773	23.7%	90,573	11.7%
Czechia	541,638	5.9%	61,863	18.2%
Hungary	541,616	17.8%	104,742	0.9%
Poland	2,411,708	30.8%	376,571	0.7%
Romania	1,212,488	43.8%	143,222	10.7%
Slovakia	362,790	39.6%	41,318	17.8%
**NE**	**1,014,344**	**18.7%**	**172,620**	**-1.2%**
Denmark	56,052	6.4%	10,464	-0.2%
Estonia	76,163	22.5%	20,775	2.5%
Finland	160,931	8.4%	9,390	8.8%
Ireland	99,775	11.2%	17,623	-1.2%
Latvia	151,472	23.3%	37,827	-1.7%
Lithuania	256,382	33.5%	63,197	-1.0%
Sweden	137,362	10.7%	2,233	-44.5%
Norway	76,207	11.8%	11,112	-7.6%
**SE**	**5,142,471**	**11.2%**	**454,329**	**0.1%**
Croatia	205,645	19.7%	13,763	16.7%
Cyprus	16,620	11.5%	4,634	0.5%
Greece	411,050	1.4%	34,118	2.4%
Italy	2,683,375	13.3%	193,036	-1.4%
Malta	4,936	32.5%	608	28.9%
Portugal	269,687	12.1%	36,851	-3.8%
Slovenia	64,121	16.0%	7,492	14.1%
Spain	1,487,037	8.5%	163,827	0.0%
**WE**	**4,763,537**	**13.1%**	**551,858**	**2.4%**
Austria	267,808	14.4%	22,662	13.3%
Belgium	325,456	-0.3%	34,570	14.1%
France	1,614,157	6.3%	144,116	13.1%
Germany	2,010,509	18.9%	285,718	-2.6%
Luxembourg	-5,296	12.2%	-793	69.4%
Netherlands	550,903	19.0%	65,584	-7.9%
**Total**	**16,695,365**	**15.5%**	**1,997,095**	**2.2%**

Notes: Columns show eYLL and eYPPLL in the base scenario, and the relative deviation (%) under two alternative assumptions: (i) LE_2050 – projected life expectancy in 2050; (ii) 20-64 – productive life restricted to ages 20–64 years. Values in the % columns represent proportional changes relative to the base scenario for each country and region for all ages and both sexes combined.

Table S12. Cross-country comparison of excess deaths estimates from major international studies and this analysis

	2020			2020-2021							2020-2023	
Country	Alicandro [1]	Islam [2]	This analysis	Karlinsky [3]	Economist [4]	Lancet [5]	WHO [6]	Levitt age-adjusted [7]	Levitt not age-adjusted [7]	This analysis	Pizzato [8]	This analysis
**CEE**												
Bulgaria	16,717	N.A	16,315	N.A	N.A	N.A	N.A	N.A	N.A	57,554	72,328	60,936
Czechia	17,264	14,400	15,796	41,480	43,942	49,100	37,040	34,079	43,262	43,501	59,014	52,003
Hungary	N.A	16,600	9,920	35,811	41,714	53,800	36,499	27,813	36,090	34,525	43,325	39,109
Poland	68,816	60,100	62,044	157,247	171,806	214,000	157,531	149,722	182,454	172,028	223,735	213,041
Romania	N.A	N.A	34,778	N.A	N.A	N.A	N.A	N.A	N.A	109,394	N.A	127,384
Slovakia	N.A	4,400	4,893	24,131	25,538	25,400	24,320	18,662	23,786	24,050	31,984	31,927
**NE**												
Denmark	-161	-160	-373	913	2,453	10,400	3,716	-3,157	2,390	957	8,882	4,695
Estonia	433	670	286	3,172	3,774	5,630	3,374	2,675	3,346	3,361	8,006	5,846
Finland	825	1,000	714	2,662	4,469	8,780	2,858	-716	4,345	3,132	17,172	16,063
Ireland	N.A	N.A	477	N.A	N.A	N.A	N.A	N.A	N.A	2,450	N.A	6,735
Latvia	1,032	820	961	6,979	7,851	12,400	7,668	6,046	7,023	7,893	13,797	12,466
Lithuania	4,643	6,800	5,161	16,008	17,396	20,000	17,253	11,283	12,274	13,969	23,813	17,455
Norway	N.A	-70	7,082	1,101	1,986	742	-100	-2,994	-182	8,227	6,970	13,435
Sweden	N.A	9,300	-362	9,926	11,976	18,100	11,253	-367	3,666	411	7,857	6,863
**SE**												
Croatia	N.A	N.A	4,771	16,826	19,186	22,900	17,178	12,205	16,050	15,834	23,115	22,802
Cyprus	N.A	N.A	31	N.A	N.A	N.A	N.A	N.A	N.A	682	N.A	1,145
Greece	N.A	7,700	5,632	24,177	25,269	25,400	19,394	20,515	29,551	23,531	38,164	42,500
Italy	99,195	89,100	96,928	167,816	190,872	259,000	160,800	115,690	166,373	158,329	227,736	266,462
Malta	N.A	N.A	263	N.A	N.A	N.A	N.A	N.A	N.A	534	N.A	917
Portugal	N.A	8,500	9,197	20,677	24,530	40,400	20,449	16,286	25,602	18,793	37,310	28,717
Slovenia	3,298	3,200	3,092	4,953	5,492	6,980	5,584	3,944	5,617	5,376	9,706	7,009
Spain	69,058	84,100	68,139	102,991	115,685	162,000	103,935	68,720	95,964	93,481	105,561	136,756
**WE**												
Austria	6,464	6,800	6,815	15,261	16,877	18,300	11,941	13,007	15,343	13,509	37,472	24,670
Belgium	N.A	17,900	17,016	20,613	23,364	32,800	17,919	13,958	19,036	21,032	28,473	32,324
France	N.A	43,500	48,415	78,910	97,390	155,000	81,849	57,767	96,831	89,948	156,511	156,840

Germany	10,164	25,900	20,705	88,446	113,242	203,000	194,987	54,740	128,557	64,619	218,111	164,382
Luxembourg	N.A	N.A	217	57	314	1,070	69	109	171	262	795	4
Netherlands	15,560	15,300	12,954	28,495	33,017	45,500	29,213	17,969	32,020	27,288	46,498	47,548

N.A – not available.

References for Table S12

1. Alicandro G, La Vecchia C, Islam N, Pizzato M. A comprehensive analysis of all-cause and cause-specific excess deaths in 30 countries during 2020. Eur J Epidemiol. 2023 Nov;38(11):1153-1164. doi: 10.1007/s10654-023-01044-x. Epub 2023 Sep 8. PMID: 37684387; PMCID: PMC10663248.
2. Islam N, Shkolnikov VM, Acosta RJ, Klimkin I, Kawachi I, Irizarry RA, Alicandro G, Khunti K, Yates T, Jdanov DA, White M, Lewington S, Lacey B. Excess deaths associated with covid-19 pandemic in 2020: age and sex disaggregated time series analysis in 29 high income countries. BMJ. 2021 May 19;373:n1137. doi: 10.1136/bmj.n1137. PMID: 34011491; PMCID: PMC8132017.
3. Karlinsky A, Kobak D. Tracking excess mortality across countries during the COVID-19 pandemic with the World Mortality Dataset. Elife. 2021 Jun 30;10:e69336. doi: 10.7554/eLife.69336. PMID: 34190045; PMCID: PMC8331176.
4. Estimated excess death count from the Economist (as of Dec 31, 2021), in https://ourworldindata.org/excess-mortality-covid.
5. COVID-19 Excess Mortality Collaborators. Estimating excess mortality due to the COVID-19 pandemic: a systematic analysis of COVID-19-related mortality, 2020-21. Lancet. 2022 Apr 16;399(10334):1513-1536. doi: 10.1016/S0140-6736(21)02796-3. Epub 2022 Mar 10. Erratum in: Lancet. 2022 Apr 16;399(10334):1468. doi: 10.1016/S0140-6736(22)00621-3. PMID: 35279232; PMCID: PMC8912932.
6. World Health Organization, Global Excess Deaths Associated with COVID-19, January 2020–December 2021. In: https://www.who.int/data/stories/global-excess-deaths- associated-with-covid-19-january-2020-december-2021.
7. Levitt M, Zonta F, Ioannidis JPA. Comparison of pandemic excess mortality in 2020-2021 across different empirical calculations. Environ Res. 2022 Oct;213:113754. doi: 10.1016/j.envres.2022.113754. Epub 2022 Jun 24. PMID: 35753371; PMCID: PMC9225924.
8. Pizzato M, Gerli AG, La Vecchia C, Alicandro G. Impact of COVID-19 on total excess mortality and geographic disparities in Europe, 2020-2023: a spatio-temporal analysis. Lancet Reg Health Eur. 2024 Jul 3;44:100996. doi: 10.1016/j.lanepe.2024.100996. Erratum in: Lancet Reg Health Eur. 2024 Oct 30;47:101117. doi: 10.1016/j.lanepe.2024.101117. PMID: 39410937; PMCID: PMC11473197.

Missing data imputation

In the Eurostat database[1], three different patterns of incomplete data were identified:

1. A single missing value in interior years (e.g. no mortality data in 2008 in the 67-year-old population[2]).
2. A single data point missing in the extreme (the first or last) year of the dataset (2002 or 2019).
3. Missing two or more data points in interior years (e.g. no mortality data in 2003-2007 in the 83-year-old population).

Depending on the pattern of missing data, we implemented two different strategies to impute missing observations.

For pattern 1, a single missing point bounded by observed years, we imputed an average value from the preceding and following years (simple linear interpolation).

For patterns 2 and 3, we relied on year-to-year percentage changes from the nearest age band for which the data were complete. For example, for Hungary, population counts for each age between 85 and 89 and both sexes were available for the years 2002-2006 and 2012-2023. For the missing 2007-2011 period, we used year-to-year percentage changes of the sex-specific population counts of those aged 84. We computed changes backwards (from 2012 to previous years). An alternative approach would be to compute changes using forward (from 2006 onwards) year-to-year changes. However, the use of forward changes would not be feasible for some ages (90+) as missing data were found in the first eleven years of the period analysed. Therefore, to be consistent, we opted for the backwards changes in all imputations.

The formula used for patterns 2 and 3 and for the example discussed above referring to 85 year old population:

$$x_{t-1}^{85} = x_t^{85} \times \frac{x_{t-1}^{84}}{x_t^{84}}$$

where:

x_t^{85}– first available value after the gap for the 85 years old series,

[1] https://ec.europa.eu/eurostat/databrowser/view/demo_pjan/default/table?lang=en

[2] In our analyses, we used 5-year age groups; however, the source data on the number of deaths and population are reported for each single age. We aggregated these to 5-year age groups.

x^{85}_{t-1} – missing value for the 85 years old in the preceding year,

$x^{84}_{t}$and x^{84}_{t-1} – observed counts for the reference age (84 years) that contains no missing data points,

$\frac{x^{84}_{t-1}}{x^{84}_{t}}$- – the year-to-year growth rate from the complete series (aged 84) carried over to the series of aged 85.

Additionally, we identified an odd pattern of Hungary population counts for the “Open-ended age class” that reports deaths among those aged 99+. During the first five years (2002-2006), the population in this class increased from 38,007 to 42,236 (both sexes combined), while in 2007, the respective count more than tripled to 135,523 and increased further to 169,759 in 2011. In 2012, the figure declined to 13,643; in 2013 it was 1,255, and remained <5,000 until 2023. These inconsistencies precluded us from using the figures; instead, we relied on the linear interpolation of aggregated 85+ aged population counts for this country, using data from periods for which the counts were more credible (2002-2006 and 2012-2023). For this reason, the estimates of excess mortality measures for Hungary for the age group 85+ should be treated with caution. Data from other countries was free from such concerns.

The data coverage (percentage of dataset completeness) for each country and details on missing data are reported in Table S10.

Missing data imputations were carried out using Microsoft Excel 2021.

Table S13. Details of data coverage

Country	Completeness of data [%]	Main patterns of missing data
Austria, Belgium, Bulgaria, Cyprus, Czechia, Denmark, Estonia, Finland, Greece, Italy, Latvia, Poland, Portugal, Romania, Slovakia, Slovenia, Spain, Sweden, Norway	100	-
Germany	98.85	Data missing for those aged 95+ in 2002-2010, only in the population data
Ireland	99.88	Data missing for those aged 99 in 2002-2006, only in the population data
France	99.93	Single missing values for those aged 99, only in the population data
Croatia	99.63	Data missing for those aged 85+ in 2012, only in the population data
Lithuania	97.65	Data missing for 2013, only in population data
Luxembourg	99.31	Data missing for those aged 95+ in 2002-2008, only in the population data
Hungary	96.89	Data missing for those aged 85-89 in 2007-2011 and 90+ in 2002-2012, only in the population data
Malta	99.29	Data missing for those aged 90+ in 2003-2005, only in the population data
Netherlands	99.98	Data missing for those aged 99 in 2002, only in the mortality data
